# Robust and Fast Markov Chain Monte Carlo Sampling of Diffusion MRI Microstructure Models

**DOI:** 10.3389/fninf.2018.00097

**Published:** 2018-12-18

**Authors:** Robbert L. Harms, Alard Roebroeck

**Affiliations:** Department of Cognitive Neuroscience, Faculty of Psychology & Neuroscience, Maastricht University, Maastricht, Netherlands

**Keywords:** Markov Chain Monte Carlo (MCMC) sampling, diffusion MRI, microstructure, biophysical compartment models, parallel computing, GPU computing

## Abstract

In diffusion MRI analysis, advances in biophysical multi-compartment modeling have gained popularity over the conventional Diffusion Tensor Imaging (DTI), because they can obtain a greater specificity in relating the dMRI signal to underlying cellular microstructure. Biophysical multi-compartment models require a parameter estimation, typically performed using either the Maximum Likelihood Estimation (MLE) or the Markov Chain Monte Carlo (MCMC) sampling. Whereas, the MLE provides only a point estimate of the fitted model parameters, the MCMC recovers the entire posterior distribution of the model parameters given in the data, providing additional information such as parameter uncertainty and correlations. MCMC sampling is currently not routinely applied in dMRI microstructure modeling, as it requires adjustment and tuning, specific to each model, particularly in the choice of proposal distributions, burn-in length, thinning, and the number of samples to store. In addition, sampling often takes at least an order of magnitude, more time than non-linear optimization. Here we investigate the performance of the MCMC algorithm variations over multiple popular diffusion microstructure models, to examine whether a single, well performing variation could be applied efficiently and robustly to many models. Using an efficient GPU-based implementation, we showed that run times can be removed as a prohibitive constraint for the sampling of diffusion multi-compartment models. Using this implementation, we investigated the effectiveness of different adaptive MCMC algorithms, burn-in, initialization, and thinning. Finally we applied the theory of the Effective Sample Size, to the diffusion multi-compartment models, as a way of determining a relatively general target for the number of samples needed to characterize parameter distributions for different models and data sets. We conclude that adaptive Metropolis methods increase MCMC performance and select the Adaptive Metropolis-Within-Gibbs (AMWG) algorithm as the primary method. We furthermore advise to initialize the sampling with an MLE point estimate, in which case 100 to 200 samples are sufficient as a burn-in. Finally, we advise against thinning in most use-cases and as a relatively general target for the number of samples, we recommend a multivariate Effective Sample Size of 2,200.

## 1. Introduction

**Figure 1:**
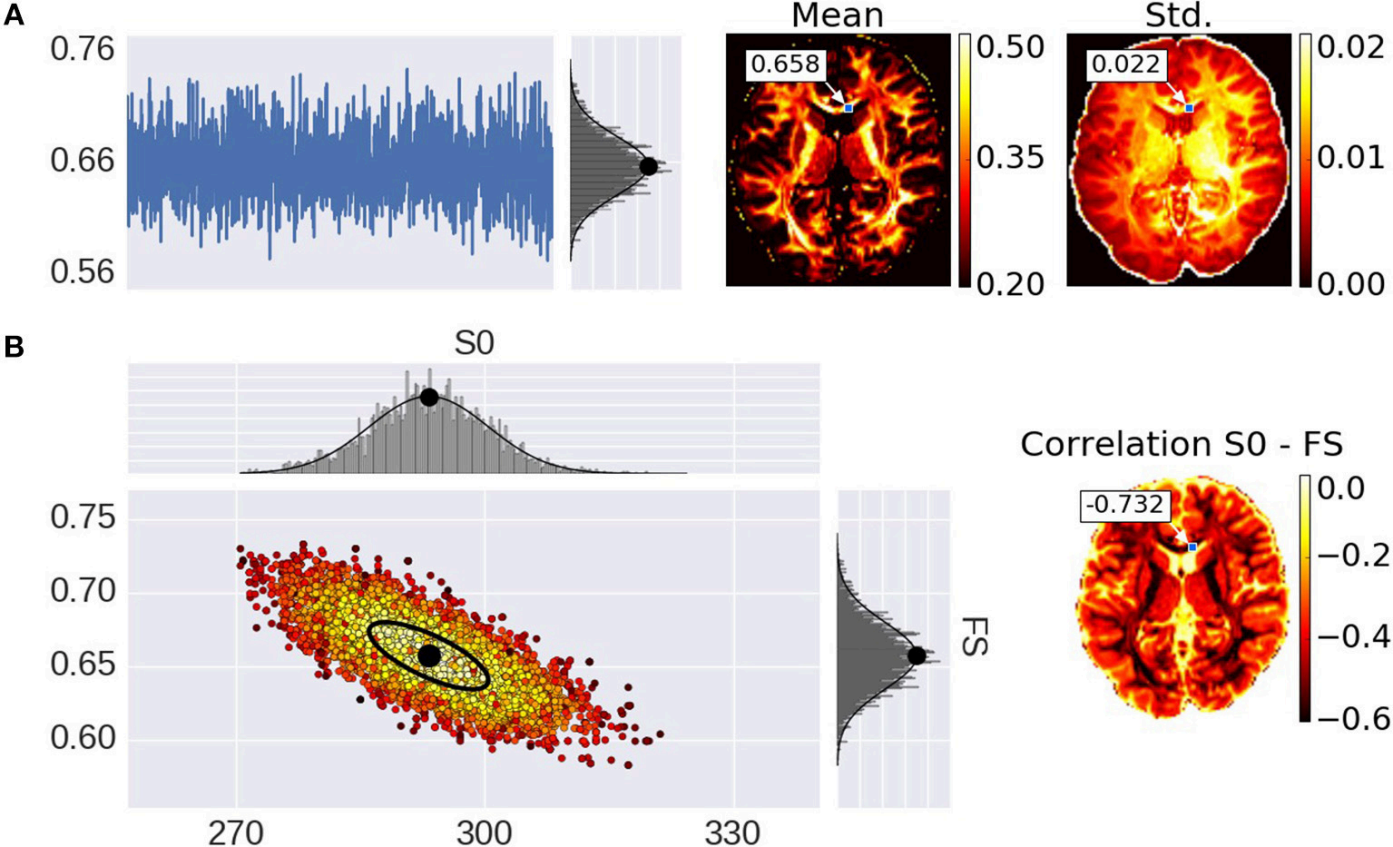
Illustration of parameter uncertainty and correlation for the Ball&Stick model using MCMC sampling, with the Fraction of Stick (FS) and the non-diffusion weighted signal intensity (S0). **(A)** On the left, a single FS sampling trace and its corresponding histogram for the highlighted voxel with a Gaussian distribution function fitted to the samples with its mean indicated by a black dot. On the right, the mean and standard deviation (std.) maps generated from the independent voxel chains per voxel. **(B)** On the left, the scatter-plot for two parameters (FS and S0) with the corresponding marginal histograms for the voxel highlighted in the maps. On the right, the S0-FS correlation map.

Advances in microstructure modeling of diffusion Magnetic Resonance Imaging (dMRI) data have recently gained popularity as they can obtain a greater specificity than Diffusion Tensor Imaging (DTI), in relating the dMRI signal to the underlying cellular microstructure, such as axonal density, orientation dispersion, or diameter distributions. Typically, dMRI models are fitted to the data using non-linear optimization (Assaf et al., 2004, 2008, 2013; Assaf and Basser, 2005; Panagiotaki et al., 2012; Zhang et al., 2012; Fieremans et al., 2013; De Santis et al., 2014a,b; Jelescu et al., 2015; Harms et al., 2017), linear convex optimization (Daducci et al., 2015), stochastic optimization (Farooq et al., 2016), or analytical (Novikov et al., 2016) methods to obtain a parameter point estimate per voxel. These point estimates provide scalar maps over the brain, of micro-structural parameters such as the fraction of restricted diffusion as a proxy for fiber density. These point estimates however, do not provide the entire posterior distribution, which can be useful in probabilistic tractography and to quantify the uncertainty and interdependency of parameters, for example. The gold standard of obtaining the posterior distribution is by using Markov Chain Monte Carlo (MCMC) sampling, as for example in (Behrens et al., 2003; Alexander, 2008; Alexander et al., 2010; Sotiropoulos et al., 2013). MCMC generates, per voxel, a multi-dimensional chain of samples, the stationary distribution of which is the posterior distribution, i.e., the probability density of the model parameters given the data. Per voxel, these samples capture parameter dependencies, multi modality, and the width of peaks around optimal parameter values. For instance, summarizing the chain under Gaussian assumptions with a sample co-variance matrix, would provide mean parameter estimates and corresponding uncertainties (the standard deviation), as well as inter-parameter correlations (Figure 1).

Despite the advantages of providing the full posterior information, MCMC sampling is currently not routinely applied in dMRI microstructure modeling, as it often requires an adjustment and tuning specific to each model, particularly in the choice of proposals, burn-in length, thinning, and the number of samples to store. In addition, sampling often takes at least an order of magnitude more time than non-linear optimization, even when using GPU's to accelerate the computations by one or two orders of magnitude (Hernández et al., 2013).

The main purpose of this paper is to provide an effective MCMC sampling strategy combined with an efficient GPU accelerated implementation. To this end, we investigate the performance of a few variants of Random Walk Metropolis MCMC algorithms over multiple popular diffusion microstructure models, to see whether a single well performing variation could be applied efficiently and robustly to many models. Furthermore, we discuss the use of burn-in and thinning in dMRI modeling and apply the concept of effective sample sizes, to determine a lower bound on the number of samples needed. To reduce run-time constraints we provide an efficient parallel GPU implementation of all models and MCMC algorithms in the open source Microstructure Diffusion Toolbox (MDT; https://github.com/cbclab/MDT).

## 2. Methods

The biophysical (multi-)compartment models and the Markov Chain Monte Carlo (MCMC) algorithms used in this study are implemented in a Python based GPU accelerated toolbox (the Microstructure Diffusion Toolbox, MDT, freely available under an open source L-GPL license at https://github.com/cbclab/MDT). Its modular design allows arbitrary combinations of models with likelihood and prior distributions. The MCMC implementations are voxel-wise parallelized using the OpenCL framework, allowing parallel computations on multi-core CPU and/or Graphics Processing Units (GPUs).

We used the models and MCMC routine as implemented in MDT version 0.15.0. Unless stated otherwise, we initialized the MCMC sampling with a Maximum Likelihood Estimator (MLE) obtained from non-linear parameter optimization using the Powell routine with cascaded model initialization and patience 2 (Harms et al., 2017). Scripts for reproducing the results in this article can be found at https://github.com/robbert-harms/sampling_paper.

First, we defined and reviewed posteriors, likelihoods, and priors relevant to diffusion multi-compartment models. We next defined the Metropolis-Hastings as the general type of Markov Chain Monte Carlo algorithms used in this work. Then, under the assumptions of the symmetric and current position centered proposals, updated one dimension at a time, we derived the Metropolis-Within-Gibbs algorithm. The Metropolis-Within-Gibbs algorithm is then explained with and without the use of adaptive proposals. We subsequently defined burn-in, thinning, effective sample size, and number of samples as the targets of the investigation of the diffusion microstructure models.

### 2.1. Diffusion Microstructure Models

The general multi-compartment diffusion microstructure model has the form of a weighted sum of single compartments:

(1) $$\begin{array}{r} \text{S}=S_{0}\overset{n}{\underset{i=0}{\sum }}w_{i}{\text{S}}_{i} \end{array}$$

Where *S*_0_ is the signal for the non-diffusion weighted (or b0) acquisitions, *w*_*i*_ the volume fractions (signal weights, signal fractions, or water fractions) and S_*i*_ is the signal function for the *i*′th of *n* total compartments. For this work we selected the Tensor (Basser et al., 1994), Ball&Sticks (Behrens et al., 2003), NODDI (Zhang et al., 2012), and CHARMED (Assaf et al., 2004) models. Table 1 shows these multi-compartment models (henceforth simply “models”), their constituent compartments and total number of parameters including estimation of *S*_0_. For signal model naming we use the postfix “_in[n]” to identify the number of restricted compartments employed in models which allow multiple intra-axonal compartments. For example, CHARMED_in2 indicates a CHARMED model with 2 intra-axonal compartments (and the regular single extra-axonal compartment), for each of two unique fiber orientations in a voxel. Table 2 lists the compartments referenced to in Table 1, with the corresponding optimizable parameters listed in Table 3. See (Harms et al., 2017) for implementation details of these compartments and multi-compartment models.

**Table 1:** The used composite multi-compartment models, their compartments (divided into intra-, extra-axonal, and isotropic) and total number of parameters.

**Model**	**Restricted (*in*tra-cellular) compartments **	**Hindered (*ex*tra-cellular) compartments **	**Isotropic compartments **	**Number of parameters**	**Acquisition requirements**
Tensor	–	Tensor	–	7	*b* < 1.5·10^6^*s*/*m*^2^
Ball&Sticks _in[*n*]	Stick (*n*-times)	–	Ball	1+3*n*	–
NODDI	NODDI_in	NODDI_ex	Ball	6	≥2 b-values / shells
CHARMED _in[*n*]	CHARMED _in (*n*-times)	Tensor	–	7+4*n*	≥2 b-values / shells, $b_{\text{max}}\geq 4.0\cdot 10^{6}s/m^{2}$

*For an overview of the individual compartments, please see Table 2*.

**Table 2:** The single compartment models, see Table 3 for an overview of the optimizable parameters.

**Compartment**	**Signal function**	**Compartment model parameters**
Tensor	$\text{S}=e^{-b\left(d_{∥}{\left(\text{n}\cdot \text{g}\right)}^{2}+d_{{⊥}_{1}}{\left({\text{n}}_{{⊥}_{1}}\cdot \text{g}\right)}^{2}+d_{{⊥}_{2}}{\left({\text{n}}_{{⊥}_{2}}\cdot \text{g}\right)}^{2}\right)}$ **n**_⊥_1__ = rotate(**n**, ψ) **n**_⊥_2__ = **n**×**n**_⊥_1__	*d*_∥_, *d*_⊥_1__, *d*_⊥_2__, θ, ϕ, ψ
Ball	**S** = *e*^−*bd*^	*d*
Stick	**S** = *e*^−*bd*^(**n**·**g**)^2^	*d*, θ, ϕ
NODDI_in	$\text{S}=\underset{S^{2}}{\int }f\left(\text{n},\kappa \right)e^{-bd{\left(\text{n}\cdot \text{g}\right)}^{2}}d\text{n}$	*d*, θ, ϕ, κ
NODDI_ex	$\text{S}=e^{-b{\text{g}}^{⊺}\left(\underset{S^{2}}{\int }f\left(\text{n},\kappa \right)D\left(\text{n}\right)d\text{n}\right)\text{g}}$	*d*_∥_, *d*_⊥_, θ, ϕ, κ
CHARMED_in	$\text{S}=\overset{\text{N}}{\underset{i=1}{\sum }}v_{i}\left[{\text{S}}_{∥}\left(\text{q},\Delta \right)\cdot {\text{S}}_{{⊥}_{i}}\left(\text{q},\text{TE}\right)\right]$ ${\text{S}}_{∥}\left(\text{q},\Delta \right)=e^{-4\pi^{2}|\text{q}{|}^{2}{\left(\text{n}\cdot \text{g}\right)}^{2}\left(\Delta -\delta /3\right)d}$ ${\text{S}}_{{⊥}_{i}}\left(\text{q},\text{TE}\right)=e^{-\left[4\pi^{2}|\text{q}{|}^{2}\left(1-{\left(\text{n}\cdot \text{g}\right)}^{2}\right){\text{R}}_{i}^{4}/\left(\frac{d\cdot \text{TE}}{2}\right)\right]\cdot \left(\frac{7}{96}\right)\cdot \left[2-\left(\frac{99}{112}\right){\text{R}}_{i}^{2}/\left(\frac{d\cdot \text{TE}}{2}\right)\right]}$	*d*, θ, ϕ

*The primary direction of diffusivity **n**, is parameterized using polar coordinates with angles θ, ϕ and radius d. The variables b, **g**, **q**, Δ, δ, G, and TE are sequence settings. In the Tensor compartment, the function rotate(n, ψ) rotates the Tensor around **n** by the angle ψ. In the NODDI models, the function f(**n**, κ)d**n** gives the probability of finding fiber bundles along orientation **n** using a Watson distribution with parameter κ integrated over the unit sphere S^2^. In the NODDI_ex model, the diffusion tensor D(**n**) is defined as a cylindrically symmetric Tensor (alike the Tensor in this table except for the symmetry). In the CHARMED_in compartment N is the number of gamma cylinders used, v_i_ is the weight per gamma distributed cylinders and R_i_ is the radius per cylinder. In previous work |**q**|^2^(**n**·**g**)^2^ is sometimes denoted as $|{\text{q}}_{∥}{|}^{2}$ and |**q**|^2^(1−(**n**·**g**)^2^) as $|{\text{q}}_{⊥}{|}^{2}$, we inlined these identities here in the CHARMED_in equation*.

**Table 3:** The parameter descriptions corresponding to the diffusion compartment models in Table 2.

**Parameter**	**Compartments**	**Usage**
*d*_∥_ (or *d*)	Tensor, Ball, Stick, NODDI_in, NODDI_ex, CHARMED_in	Parallel diffusivity along the primary direction of diffusion **n**
*d*_⊥_1__	Tensor	Perpendicular diffusivity, perpendicular to both *d*_∥_ and *d*_⊥_2__.
*d*_⊥_2__	Tensor	Perpendicular diffusivity, perpendicular to both *d*_∥_ and *d*_⊥_2__.
θ	Tensor, Stick, NODDI_in, NODDI_ex, CHARMED_in	Polar angle used to parameterize **n**, the primary direction of diffusion
ϕ	Tensor, Stick, NODDI_in, NODDI_ex, CHARMED_in	Azimuth angle used to parameterize **n**, the primary direction of diffusion
ψ	Tensor	Used to rotate the Tensor around its primary axis
κ	NODDI_in, NODDI_ex	The dispersion index of the Watson distribution

### 2.2. Posterior, Likelihoods and Priors

Given observations O and a model with parameters **x**∈ℝ^*n*^, we can construct a posterior distribution p(**x**|O) from a log-likelihood distribution *l*(O|**x**) and prior distribution *p*(**x**), as:

(2) $$\begin{array}{r} \ln\text{ p}\left(\text{x}|O\right)\propto l\left(O|\text{x}\right)+\ln\;p\left(\text{x}\right) \end{array}$$

In this work we are interested in approximating the posterior density of p(**x**|O) using MCMC sampling.

#### 2.2.1. Likelihood Distribution

The likelihood distribution *l*(O|**x**) contains a signal model, embedding the diffusion microstructure modeling assumptions combined with a noise model. As discussed in previous work (Alexander, 2009; Panagiotaki et al., 2012; Harms et al., 2017), we use the Offset Gaussian model as likelihood distribution:

(3) $$\begin{array}{r} l\left(O|\text{x}\right)=-\frac{\sum \left(O-\sqrt{\text{S}{\left(\text{x}\right)}^{2}+\sigma^{2}}\right)}{2\sigma^{2}}-m\cdot \log\left(\sigma \sqrt{2\pi }\right) \end{array}$$

with *l*(O|**x**) the log-likelihood function, **x** the parameter vector, O the observations (the data volumes), **S**(**x**) the signal model, σ the standard deviation of the Gaussian distributed noise (of the complex valued data, i.e., before calculation of magnitude data), and *m* the number of volumes in the dataset (number of observations). We estimated σ a priori from the reconstructed magnitude images using the σ_**mult**_ method in Dietrich et al. (2007, Equation A6).

#### 2.2.2. Priors

The prior distribution *p*(**x**) describes the a priori knowledge we have about the model and its parameters. We construct a complete model prior as a product of priors per parameter, *p*_*i*_(**x**_*i*_) (see Appendix Table 1), with one or more model specific priors over multiple parameters, *p*_*j*_(**x**|*M*), for model prior *j* of model *M* (see Appendix Table 2):

(4) $$\begin{array}{r} p\left(\text{x}\right)=\prod p_{i}\left({\text{x}}_{i}\right)\cdot \prod p_{j}\left(\text{x}|M\right) \end{array}$$

Assuming no further a priori knowledge than logical or biologically plausible ranges, we used uniform priors for each parameter, *p*_*i*_(**x**_*i*_)~*U*(*a, b*). Additionally, for multi-compartment models with volume fraction weighted compartments (i.e., Ball&Stick_in[*n*], NODDI, and CHARMED_in[*n*]) we added a prior on the *n*−1 volume fractions *w*_*k*_ to ensure $\overset{n-1}{\underset{k=0}{\sum }}w_{k}<=1$, to ensure proper volume fraction weighting. Note that the last volume fraction was not sampled but was set to one minus the sum of the others, $w_{n}=1-\overset{n-1}{\underset{k=0}{\sum }}w_{i}$. To the Tensor compartment (used in the Tensor and CHARMED_in1 model), to ensure strictly decreasing diffusivities (*d*>*d*_⊥_0__>*d*_⊥_1__), this prevents parameter aliasing of the Tensor orientation parameters [see (Gelman et al., 2013) on aliasing].

### 2.3. Markov Chain Monte Carlo

Markov Chain Monte Carlo (MCMC) is a class of numerical approximation algorithms for sampling from the probability density function π(·) of a target random variate, by generating a Markov chain **X**^(0)^, **X**^(1)^, … with stationary distribution π(·). There are a large number of MCMC algorithms, including Metropolis-Within-Gibbs (a.k.a Metropolis) (Metropolis et al., 1953), Metropolis-Hastings (Hastings, 1970), Gibbs (Turchin, 1971; Geman and Geman, 1984), Component-wise Hit-And-Run Metropolis (Turchin, 1971; Smith, 1984), Random Walk Metropolis (Muller, 1994), Multiple-Try Metropolis Liu et al. (2000), No-U-Turn sampler (Hoffman and Gelman, 2011), and many more. All of these algorithms are known as special cases of the Metropolis-Hastings algorithm and differ only in the proposal distributions they employ (Chib and Greenberg, 1995; Johnson et al., 2013).

The general Metropolis-Hastings algorithm works as follows. Given a current position **X**^(*t*)^ at step *t* on a *p*-dimensional Markov chain, a new position **X**^(*t*+1)^ is obtained by generating a candidate position **Y** from the proposal density *q*(**X**^(*t*)^|·), which is then either accepted with probability α, or rejected with probability 1−α. If the candidate position is accepted, **X**^(*t*+1)^ = **Y**, else, **X**^(*t*+1)^ = **X**^(*t*)^. The acceptance criteria α is a function given by Hastings (1970):

(5) $$\begin{array}{r} \alpha \left({\text{X}}^{\left(t\right)},\text{Y}\right)=\min\left(1,\frac{\pi \left(\text{Y}\right)}{\pi \left({\text{X}}^{\left(t\right)}\right)}\;\frac{q\left({\text{X}}^{\left(t\right)}|\text{Y}\right)}{q\left(\text{Y}|{\text{X}}^{\left(t\right)}\right)}\right) \end{array}$$

where *π*(·) is our target density, generally given by our posterior distribution function p(**X**|·). The subsequent collection of points {**X**^(0)^, …, **X**^(*s*)^} for a sample size *s* is called the chain and is the algorithm's output. The *ergodic* property of this algorithm guarantees that this chain converges (in the long run) to a stationary distribution which approximates the target density function *π*(·) (Metropolis et al., 1953; Hastings, 1970).

In this work we use a component wise updating scheme in which a new position **X**^(*t*+1)^ is proposed one component (i.e., one dimension) at a time, in contrast to updating all *p* dimensions at once. Combined with a symmetric proposal distribution centered around the current sampling position (for every component), this scheme is typically referred to as Metropolis-Within-Gibbs (MWG; Sherlock et al., 2010; Robert, 2015; van Ravenzwaaij et al., 2016). Let ${\text{X}}^{\left(t\right)}=\left({\text{X}}_{0}^{\left(t\right)},\ldots ,{\text{X}}_{p}^{\left(t\right)}\right)$ define the components ${\text{X}}_{i}^{\left(t\right)}$ of **X**^(*t*)^, then we can define

$$\begin{array}{l} {\text{Y}}_{i}=\left({\text{X}}_{0}^{\left(t+1\right)},\ldots ,{\text{X}}_{i-1}^{\left(t+1\right)},{\text{Y}}_{i}^{*},{\text{X}}_{i+1}^{\left(t\right)},\ldots ,{\text{X}}_{p}^{\left(t\right)}\right) \end{array}$$

as the candidate position for component *i*, and

$$\begin{array}{l} {\text{X}}^{{\left(t+1\right)}^{*}}=\left({\text{X}}_{0}^{\left(t+1\right)},\ldots ,{\text{X}}_{i-1}^{\left(t+1\right)},{\text{X}}_{i}^{\left(t\right)},{\text{X}}_{i+1}^{\left(t\right)},\ldots ,{\text{X}}_{p}^{\left(t\right)}\right) \end{array}$$

as the temporary position in the chain while component *i* is being updated. The proposals ${\text{Y}}_{i}^{*}$ are generated using the symmetric proposal $q_{i}\left({\text{X}}^{{\left(t+1\right)}^{*}}|\cdot \right)$ which updates the *i*th component dependent on the components already updated. One iteration of the MWG algorithm cycles through all *i* components, where each proposal **Y**_*i*_ is accepted or rejected using probability $\alpha \left({\text{X}}^{\left(t+1\right)*},{\text{Y}}_{i}\right)$.

### 2.4. Proposal Distributions

As symmetric proposal distributions for our MWG algorithm we used centered Normal distributions, i.e., $q_{i}\left({\text{X}}^{{\left(t+1\right)}^{*}}|\cdot \right)\sim N\left({\text{X}}_{i}^{\left(t\right)},\sigma_{i}\right)$, where σ_*i*_ is the proposal standard deviation of the *i*th component (not to be confused with the σ used in the likelihood distribution above). For the orientation parameter ψ we used a circular Normal modulus *π*, i.e., $q_{i}\left({\text{X}}^{{\left(t+1\right)}^{*}}|\cdot \right)\sim N\left({\text{X}}_{i}^{\left(t\right)},\sigma_{i}\right)\;\mathrm{mod}\;\pi$. The orientation parameters *θ* and *ϕ* are proposed using a standard Normal distribution, but are immediately transformed together such that the corresponding vector lies in the right hemisphere of the unit circle. See Appendix Table 3 for an overview of the default proposal distributions used per parameter.

### 2.5. Adaptive Metropolis

While in the traditional Metropolis-Within-Gibbs algorithm each σ_*i*_ in the proposal distribution is fixed, variations of this algorithm exist that auto-tune each σ_*i*_ to improve the information content of the Markov chain. While technically each of these variations is a distinct MCMC algorithm, we consider and compare three of these variations here as proposal updating strategies for the MWG algorithm.

The first adaptation strategy compared is the Single Component Adaptive Metropolis (*SCAM*) algorithm (Haario et al., 2005), which works by adapting the proposal standard deviation to the empirical standard deviation of the component's marginal distribution. That is, the standard deviation $\sigma_{i}^{\left(t\right)}$ for the proposal distribution of the *i*th component at time *t* is given by:

(6) $$\sigma_{i}^{\left(t\right)}=\{\begin{array}{ll} \sigma_{i}^{\left(0\right)}, & t\leq t_{s}\\ 2.4*(\sqrt{\text{Var}(X_{i}^{\left(0\right)},\ldots ,X_{i}^{\left(t-1\right)})}+\epsilon_{i}), & t>t_{s} \end{array}$$

where *t*_*s*_ denotes the iteration after which the adaptation starts (we use *t*_*s*_ = 100). A small constant ϵ_*i*_ is necessary to prevent the standard deviation from shrinking to zero (we use $\epsilon_{i}=10^{-5}\cdot \sigma_{i}^{\left(0\right)}$). The *SCAM* algorithm has been proven to retain ergodicity, meaning it is guaranteed to converge to the right stationary distribution in the limit of infinite samples (Haario et al., 2005).

The other two methods work by adapting the acceptance rate of the generated proposals. The acceptance rate is the ratio of accepted to generated proposals and is typically updated batch-wise. In general, by decreasing the proposal standard deviation the acceptance rate increases and vice versa. Theoretically, for single component updating schemes (like in this work), the optimal target acceptance rate is 0.44 (Gelman et al., 1996).

The first of the two acceptance rate scaling strategies is from the FSL BedpostX software package. This strategy, which we refer to as the *FSL* strategy, tunes the acceptance rate to 0.5 (Behrens et al., 2003). It works by multiplying the proposal variance by the ratio of accepted to rejected samples, i.e., it multiplies the standard deviation σ_*i*_ by $\sqrt{\left(a+1\right)/\left(b-a+1\right)}$ after every batch of size *b* with *a* accepted samples. We update the proposals after every batch of size 50 (*b* = 50) (Behrens et al., 2003). Since this method never ceases the adaptation of the standard deviations, it theoretically loses ergodicity of the chain (Roberts and Rosenthal, 2007, 2009).

The last method, the Adaptive Metropolis-Within-Gibbs (*AMWG*) method (Roberts and Rosenthal, 2009) uses the current acceptance rate over batches to tune the acceptance rate to 0.44. After the *n*th batch of 50 iterations (Roberts and Rosenthal, 2009), this method updates the logarithm of σ_*i*_ by adding or subtracting an adoption amount $\delta \left(n\right)=\sqrt{1/n}$ depending on the acceptance rate of that batch. That is, after every batch, σ_*i*_ is updated by:

(7) $$\sigma_{i}^{\left(t\right)}=\{\begin{array}{ll} \sigma_{i}^{\left(t-n\right)}\cdot \exp(\delta (n)), & {\text{ar}}_{batch}>{\text{ar}}_{target}\\ \sigma_{i}^{\left(t-n\right)}/\exp(\delta (n)), & {\text{ar}}_{batch}\leq {\text{ar}}_{target} \end{array}$$

where ar_*batch*_ is the acceptance rate of the current batch and ar_*target*_ is the target acceptance rate (0.44). Since this method features diminishing adaptation, the chain remains ergodic (Roberts and Rosenthal, 2009).

We compared all three strategies and the default, with no adaptation, on the number of effective samples they generated (see below) and on accuracy and precision, using ground truth simulation data. We sampled all models with 20,000 samples, without thinning and using the point optimized Maximum Likelihood Estimator (MLE) as a starting point. We reported statistics over the first 10,000 samples in the article, considering it is the common number of samples in MCMC sampling, and report estimates over all 20,000 samples as Supplementary Figures. Estimates of the standard error of the mean (SEM) are obtained by averaging the statistics over 10 independent MCMC runs.

### 2.6. Burn-in

Burn-in is the process of discarding the first *z* samples from the chain and using only the remaining samples in subsequent analysis. The idea is that if the starting point had a low probability then the limited number of early samples may over sample low probability regions. By discarding the first *z* samples as a burn-in, the hope is that, by then, the chain has converged to its stationary distribution and that all further samples are directly from the stationary distribution (Robert, 2015). Theoretically, burn-in is unnecessary since any empirical average

(8) $$\begin{array}{r} {\hat{\mu }}_{T}\left(g\right)=\frac{1}{T}\overset{T}{\underset{t=0}{\sum }}g\left({\text{X}}^{\left(t\right)}\right) \end{array}$$

for any function *g* will convert to *μ*(*g*) given a large enough sample size and given that the chain is ergodic (Robert, 2015). Additionally, since it can not be predicted how long it will take for the chain to reach convergence, the required burn-in can only be estimated *post-hoc*. In practice, discarding the first few thousand samples as a burn-in often works and is less time-consuming than generating a lot of samples to average out the effects of a low probability starting position.

An alternative to burn-in, or, to reduce the need for burn-in, is to use a Maximum Likelihood Estimator as starting point for the MCMC sampling (van Ravenzwaaij et al., 2016). If the optimization routine did its work well, the MLE should be part of the stationary distribution of the Markov chain, removing the need for burn-in altogether. We compare initialization using a MLE obtained using the Powell routine (Harms et al., 2017), with a initialization from a default a priori value (Appendix Table 4). For most models the MLE optimization results can be used directly, for the Tensor model we occasionally need to sort the diffusivities and reorient the *θ*, *ϕ*, and ψ angles to ensure decreasing diffusivities. To evaluate the effect of burn-in and initialization single-slice datas was sampled using the NODDI model with the default starting point and with MLE. For selected single voxels the NODDI model was also sampled using the MLE starting point and two random volume fractions as a starting point. We compare these starting points on moving mean and moving standard deviation, as well as on autocorrelation (the correlation of a chain with itself) given by:

(9) $$\begin{array}{r} R\left(s,t\right)=\frac{E\left[\left(X_{t}-\mu_{t}\right)\left(X_{s}-\mu_{s}\right)\right]}{\sigma_{t}\sigma_{s}} \end{array}$$

with mean *μ*_*t*_, variance $\sigma_{t}^{2}$ at time *t*, this computes the autocorrelation *R*(*s, t*) between times *s* and *t*.

### 2.7. Thinning

Thinning is the process of using only every *k*th step of the chain for analysis, while all other steps are discarded, with as goal reducing autocorrelation and obtaining relatively independent samples. Several authors have recommended against the use of thinning, stating that it is often unnecessary, always inefficient and reduces the precision of the posterior estimates (Geyer, 1991; MacEachern and Berliner, 1994; Jackman, 2009; Christensen et al., 2010; Link and Eaton, 2012).

The only valid reason for thinning is to avoid bias in the standard error estimate of posterior mean, when that mean estimate was computed over all (non-thinned) samples (MacEachern and Berliner, 1994; Link and Eaton, 2012). In general, thinning is only considered worthwhile if there are storage limitations, or when the cost of processing the output outweighs the benefits of reduced variance of the estimator (Geyer, 1991; MacEachern and Berliner, 1994; Link and Eaton, 2012).

To evaluate the effect of thinning we sampled a single voxel with 20,000 samples and compared the effect of using all samples in computing the posterior mean and posterior standard deviation of a volume fraction against using only a thinned amount of samples We compare the effect of taking *n* samples with a thinning of *k* (the *thinning* method) against just using all *n*·*k* samples (the *more samples* method).

### 2.8. Effective Sample Size

The Effective Sample Size (ESS) in the context of MCMC, measures the information content, or effectiveness of a sample chain. For example, 1,000 samples with an ESS of 200 have a higher information content than 2,000 samples with an ESS of 100. The ESS can be defined as the minimum size of a set of posterior samples (taken directly from the posterior), which have the same efficiency (measure of quality) in the posterior density estimation as a given chain of samples obtained from MCMC sampling (Martino et al., 2017). Conversely, ESS theory can quantify how many samples should be taken in a chain to reach a given quality of posterior estimates. We use the ESS theory to comparing proposal adaptation strategies and to estimating the minimum number of samples necessary for adequate sampling of diffusion microstructure models.

Multivariate ESS theory (Vats et al., 2015) is an extension of univariate ESS theory (Kass et al., 1998; Liu, 2004; Robert and Casella, 2004; Gong and Flegal, 2016) and computes the empirical ESS as:

(10) $$\hat{\text{ESS}}=s{\left(\frac{\left|\Lambda_{s}\right|}{\left|\Sigma_{s}\right|}\right)}^{1/p}$$

with *s* is the number of obtained samples, *p* the number of parameters, Λ_*s*_ the covariance matrix of the samples and Σ_*s*_ an estimate of the Monte Carlo standard error (the error in the chain caused by the MCMC sampling process), here calculated using a batch means algorithm (Vats et al., 2015).

### 2.9. Number of Samples

**Figure 2:**
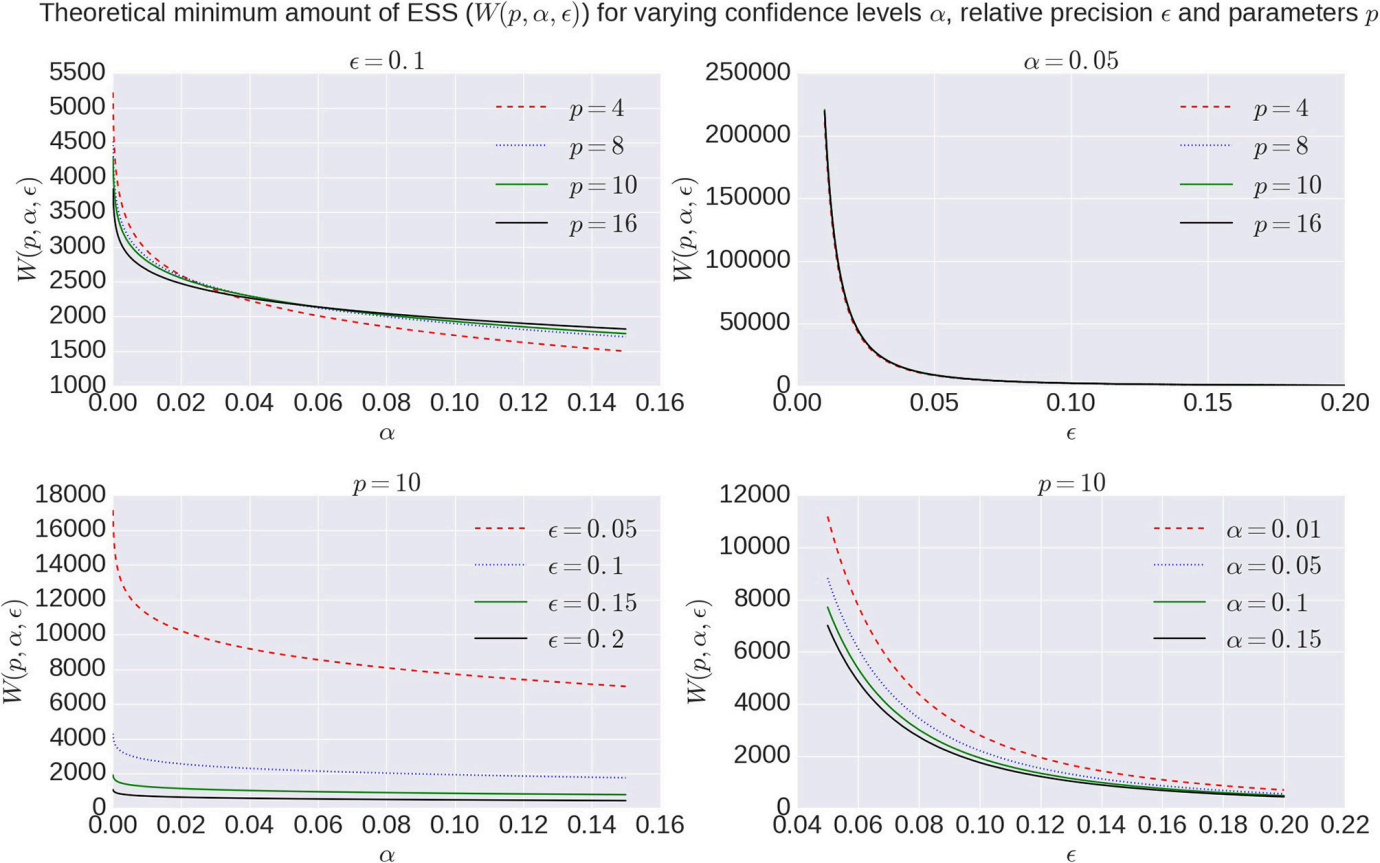
Overview of theoretical minimum ESS, *W*(*p*, α, ϵ), to reach a specific confidence level α with a desired relative precision ϵ for a model with number of parameters *p*.

The multivariate ESS theory dictates that one can terminate the sampling when the empirical number of effective samples, $\hat{\text{ESS}}$, satisfies:

(11) $$\begin{array}{r} \hat{\text{ESS}}\geq W\left(p,\alpha ,\epsilon \right) \end{array}$$

where *W*(*p*, α, ϵ) gives a theoretical lower bound with *p* the number of parameters in the model, α the level of confidence of a desired confidence region and ϵ a desired relative precision (the relative contribution of Monte Carlo error to the variability in the target distribution). *W*(*p*, α, ϵ) can be determined a priori and is defined as:

(12) $$\begin{array}{r} W\left(p,\alpha ,\epsilon \right)=\frac{2^{2/p}\pi }{{\left(p\text{Γ}\left(p/2\right)\right)}^{2/p}}\frac{\chi_{1-\alpha ,p}^{2}}{\epsilon^{2}} \end{array}$$

with χ^2^ the chi-square function and Γ(·) the Gamma function (Vats et al., 2015). Figure 2 shows the effect of α and ϵ on *W*(*p*, α, ϵ). Given the exponential increase in the number of samples need for very high confidence and precision, we aim for a 95% confidence region (α = 0.05) with a 90% precision (ϵ = 0.1) in this work.

Since online monitoring of the ESS (during MCMC sampling) is an expensive operation, and terminating on ESS will yield different sample sizes for different voxels, we instead use the ESS theory to estimate a fixed minimum number of samples needed to reach a desired ESS when averaged over a white matter mask. We sampled with the BallStick_in1, BallStick_in2, BallStick_in3, Tensor, NODDI, CHARMED_in1, CHARMED_in2, and CHARMED_in3 models, using respectively 15,000, 20,000, 25,000, 20,000, 20,000, 30,000, 40,000, and 50,000 samples and computed from those samples the average ESS over three slices in the white matter masks, namely the center slice of the volume, one slice five slices below the center and one slice five slices above. For α = 0.05 and ϵ = 0.1 we computed per model the theoretical minimum required effective sample size *W*(*p*, α, ϵ). We compared those theoretical numbers to the obtained average effective sample size and estimated a minimum required number of samples ŝ using the ratio:

(13) $$\begin{array}{r} ŝ=s+\frac{W\left(p,\alpha ,\epsilon \right)-\hat{\text{ESS}}}{\hat{\text{ESS}}/s} \end{array}$$

where *s* is the number of samples we started out with, *W*(*p*, α, ϵ) the theoretical ESS requirements and $\hat{\text{ESS}}$ the estimated number of effective samples in our chain when averaged over the white matter mask. As an estimate of computation times, we record runtime statistics for sampling the recommended number of samples using an AMD Fury X graphics card and an Intel Xeon e3 18 core CPU.

### 2.10. Datasets

For this study we used two groups of ten subjects coming from two studies, each whith a different acquisition protocol. The first ten subjects are from the freely available fully preprocessed dMRI data from the USC-Harvard consortium of the Human Connectome project. Data used in the preparation of this work were obtained from the MGH-USC Human Connectome Project (HCP) database (https://ida.loni.usc.edu/login.jsp). The data were acquired on a specialized Siemens Magnetom Connectom with 300mT/m gradient set (Siemens, Erlangen, Germany). These datasets were acquired at a resolution of 1.5 mm isotropic with Δ = 21.8ms, δ = 12.9ms, TE = 57ms, TR = 8,800ms, Partial Fourier = 6/8, MB factor 1 (i.e., no simultaneous multi-slice), in-plane GRAPPA acceleration factor 3, with 4 shells of b = 1,000, 3,000, 5,000, 10,000 s/mm^2, with respectively 64, 64, 128, 393 directions to which are added 40 interleaved b0 volumes leading to 552 volumes in total per subject, with an acquisition time of 89 min. We refer to these datasets as *HCP MGH –1.5 mm –552vol –b10k* and to the multi-shell direction table as the *HCP MGH* table. These four-shell, high number of directions, and very high maximum b- value datasets allow a wide range of models to be fitted.

The second set of ten subjects comes from the diffusion protocol pilot phase of the Rhineland Study (https://www.rheinland-studie.de) and was acquired on a Siemens Magnetom Prisma (Siemens, Erlangen, Germany) with the Center for Magnetic Resonance Research (CMRR) multi-band (MB) diffusion sequence (Moeller et al., 2010; Xu et al., 2013). These datasets had a resolution of 2.0 mm isotropic with Δ = 45.8ms, δ=16.3ms and TE = 90ms, TR = 4,500ms Partial Fourier = 6/8, MB factor 3, no in-plane acceleration with 3 shells of b = 1,000, 2,000, 3,000 s/mm^2, with respectively 30, 40, and 50 directions to which are added 14 interleaved b0 volumes leading to 134 volumes in total per subject, with an acquisition time of 10 min 21 s. Additional b0 volumes were acquired with a reversed phase encoding direction which were used to correct susceptibility related distortion (in addition to bulk subject motion) with the topup and eddy tools in FSL version 5.0.9. We refer to these datasets as *RLS-pilot – 2 mm - 134dir - b3k* and to the multi-shell direction table as the *RLS-pilot* table. These three-shell datasets represent a relatively short time acquisition protocol that still allows many models to be fitted.

Since the CHARMED_in[*n*] models require relatively high b-values (~10,000), which are not present in the RLS-pilot dataset, we will only use the HCP-MGH dataset when showing CHARMED_in[*n*] results. Additionally, since the Tensor model is only valid for b-values up to about 1,200s/mm^2, we only use the b-value 1,000s/mm^2 shell and b0 volumes during model optimization and sampling. All other models are estimated on all data volumes. For all datasets we created a white matter (WM) mask and, using BET from FSL (Smith, 2002), a whole brain mask. The whole brain mask is used during sampling, whereas averages over the WM mask are used in model or data comparisons. The WM mask was calculated by applying a lower threshold of 0.3 on the Tensor FA results, followed by a double pass 3D median filter of radius 2 in all directions. The Tensor estimate for this mask generation was calculated using a CI Ball Stick/Tensor cascade optimized with the Powell method (Harms et al., 2017).

### 2.11. Ground Truth Simulations

We performed ground truth simulations to illustrate the effects of the adaptive proposals on effective sample sizes and on accuracy and precision of parameter estimation. For all models in the study, we simulated 10,000 repeats with random volume fractions, diffusivities, and orientations, using both a HCP MGH and a RLS-pilot multi-shell direction table with Rician noise of an SNR of 30. For the Tensor model we only use the b-value 1,000 s/mm^2 shell and b0 volumes of the acquisition tables. To ensure Gaussianity of the sampled parameter distributions, we generate the parameters with a smaller range than the support of the sampling priors (Table 4). To allow a uniform SNR of 30 we fix *S*_0_ to 1·10^4^.

**Table 4:** The simulation ranges per model parameters. We generate uniformly distributed parameter values using the upper and lower bounds presented.

**Parameter**	**Lower bound**	**Upper bound**
*w*_*i*_	0.2	0.8
*d*_∥_, *d*_⊥_1__, *d*_⊥_2__	5·10^−11^	5·10^−9^
*θ*, *ϕ*, ψ	0	*π*
κ	0.1	60

Analogous to Harms et al. (2017), we compute estimation error as the mean of the (marginal) posterior minus ground truth parameter value for the intra-axonal volume fraction, i.e., fraction of stick (FS) for Ball&Sticks_in1, fraction of restricted (FR) for CHARMED_in1 and fraction of restricted (FR) for NODDI. We compute a measure of accuracy as the inverse of the mean of the average estimate error over ten thousand random repeats and a measure of precision as the inverse of the standard deviation of the average estimates. Finally, we aggregate these results per model and per experiment over 10 independent ground truth simulation trials into a mean and standard error of the mean (SEM) for both accuracy and precision. When reported, the effective sample size (ESS) is computed using the multivariate ESS theory, averaged over the 10,000 voxels with again an SEM over 10 trials.

## 3. Results

We begin by comparing the four different proposal strategies for sampling the different microstructure compartment models: Tensor, Ball&Sticks_in1, CHARMED_in1, and NODDI. We then present burn-in and thinning given an effective proposal strategy, and end with ESS estimates on the minimum number of samples needed for adequate characterization of the posterior distribution.

### 3.1. Adaptive Proposal Strategies

**Figure 3:**
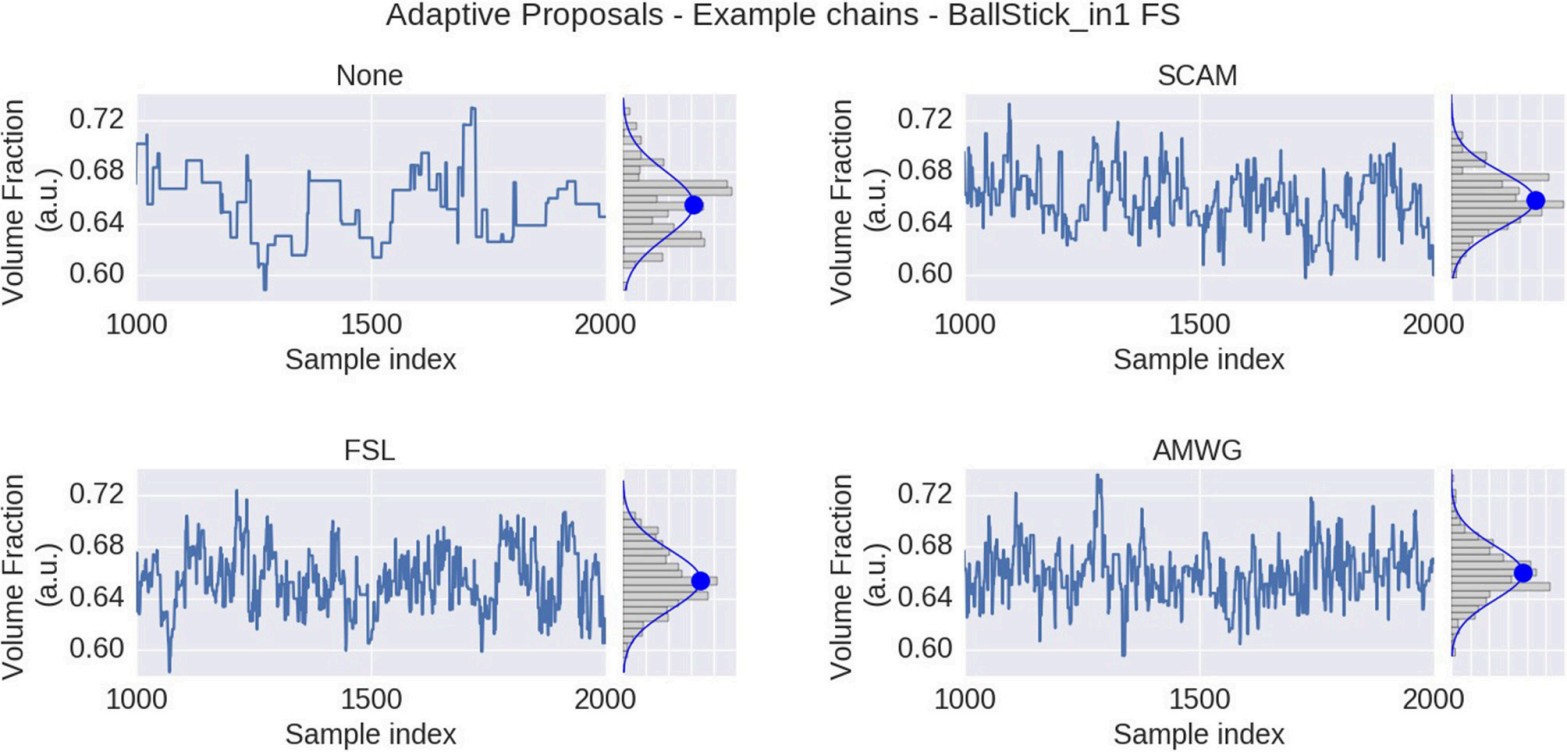
MCMC sample traces for the voxel indicated in Figure 1, using Ball&Stick_in1 Fraction of Stick (FS), for no adaptive metropolis (None), the Single Component Adaptive Metropolis (SCAM), the FSL acceptance rate scaling (FSL), and Adaptive Metropolis-Within-Gibbs (AMWG) adaptive proposal methods. Results were computed with an initial proposal standard deviation of 0.25. A Gaussian distribution function was fitted to the samples, superimposed in blue on the sample histograms, with its mean indicated by the blue dot.

We compare three different adaptive proposal strategies, the Single Component Adaptive Metropolis (SCAM), the FSL acceptance rate scaling (FSL), and the Adaptive Metropolis-Within-Gibbs (AMWG), against the default of no adaptive proposals (None). Comparisons are based on multivariate Effective Sample Size, and accuracy and precision using ground truth simulations. Figure 3 illustrates the effect of using MCMC algorithms with adaptive proposal strategies using the Ball&Stick_in1 model, the HCP MGH dataset, an initial standard deviation of 0.25, after a burn-in of 1,000 steps. The illustration clearly shows that without adaptive proposals the chain can get stuck in the same position for quite some time, while all adaptive proposal methods can adapt the standard deviations to better cover the support of the posterior distribution.

**Figure 4:**
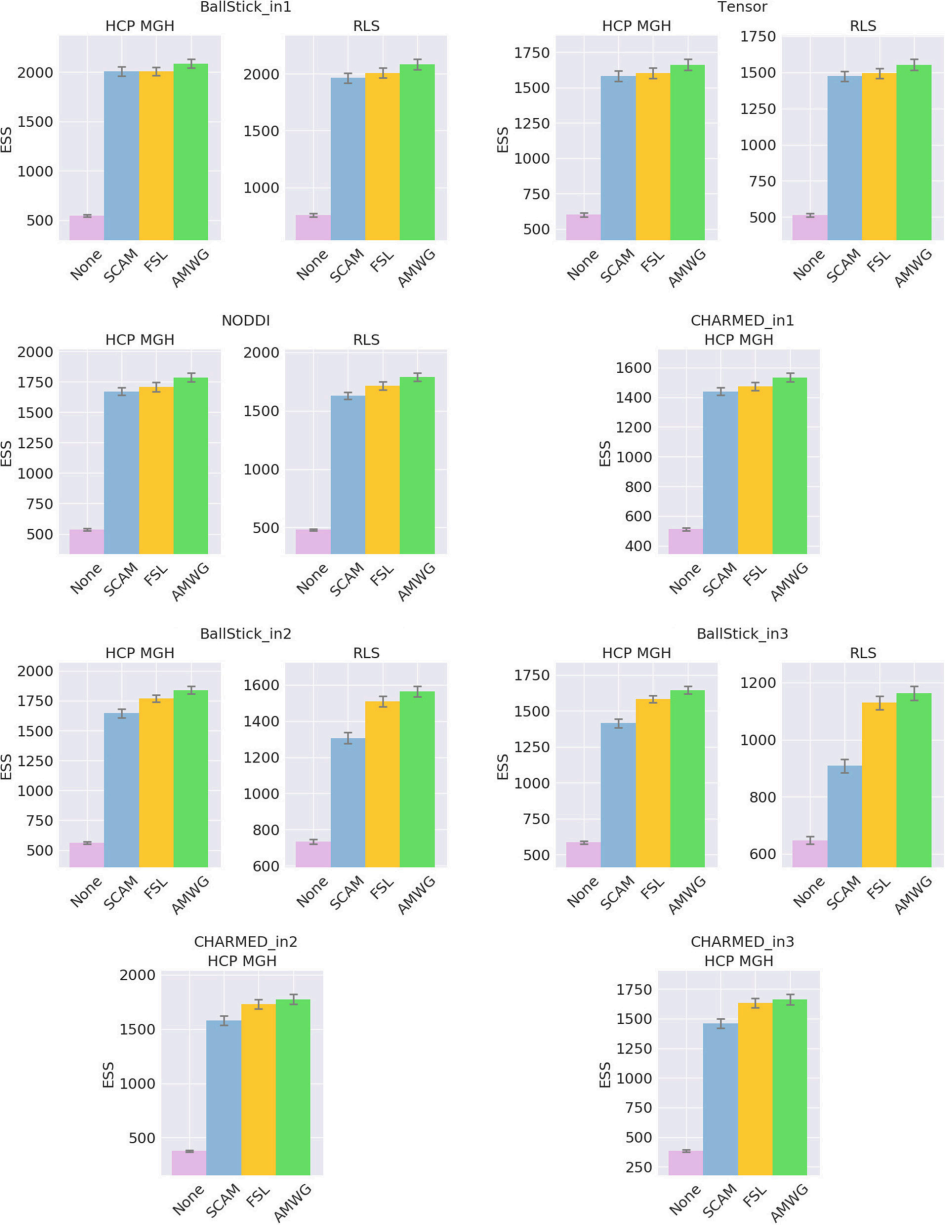
Estimated multivariate Effective Sample Size (ESS), for no adaptive metropolis (None), the Single Component Adaptive Metropolis (SCAM), the FSL acceptance rate scaling (FSL), and Adaptive Metropolis-Within-Gibbs (AMWG) adaptive proposal methods. Whiskers show the standard error of the mean computed over 10 repeats. Results are over 10,000 samples, without burn-in and thinning.

**Figure 5:**
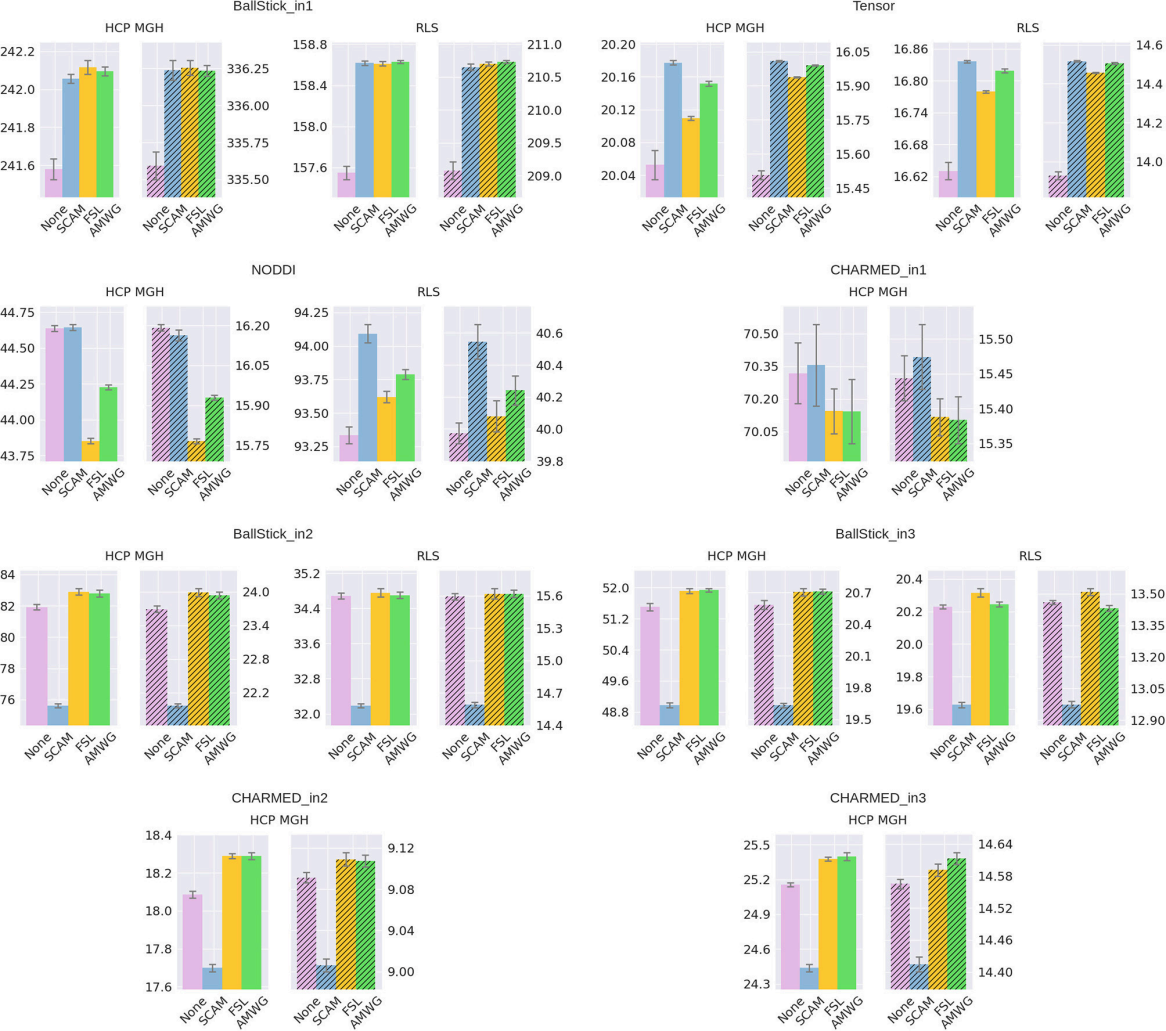
Estimated accuracy (left plots) and precision (right, shaded, plots), for no adaptive metropolis (None), the Single Component Adaptive Metropolis (SCAM), the FSL acceptance rate scaling (FSL), and Adaptive Metropolis-Within-Gibbs (AMWG) adaptive proposal methods. The results are averaged over 10,000 voxels and 10 trials, the whiskers show the standard error of the mean computed over the 10 trials. Results are over 10,000 samples, without burn-in and thinning. The reported offsets need to be added to the y-axis for absolute results.

The empirical ESS (Equation 10) measures the information content or effectiveness of a sample chain. As such, comparing the ESS for an equal number of actual samples for different proposal strategies evaluates how effectively each strategy generates useful information about the posterior distribution. Figure 4 shows that all adaptive methods clearly outperform the default, None, by generating at least 2~3 times more effective samples for equal length chains. The AMWG method generates the largest ESS in all cases, with a considerable margin with respect to SCAM and with a small margin compared to FSL, with margins increasing somewhat for more complex models and the larger HCP MGH protocol. Compared on accuracy and precision in ground truth simulations (Figure 5), the SCAM strategy performs slightly better (highest accuracy and precision) than the other adaptive methods for the lower number of parameter models (Tensor, NODDI) while the AMWG and FSL methods perform considerably better in the higher number of parameter crossing fiber models (NODDI, CHARMED_in1). Repeating, with double number of samples, the simulations of empirical ESS (Supplementary Figure 1) and accuracy and precision (Supplementary Figure 2), reproduces these results. Given the all-round efficiency, accuracy and precision, and the maintained ergodicity of the chain in the AMWG method, we selected this method to generate chains in the rest of this work.

### 3.2. Burn-in

**Figure 6:**
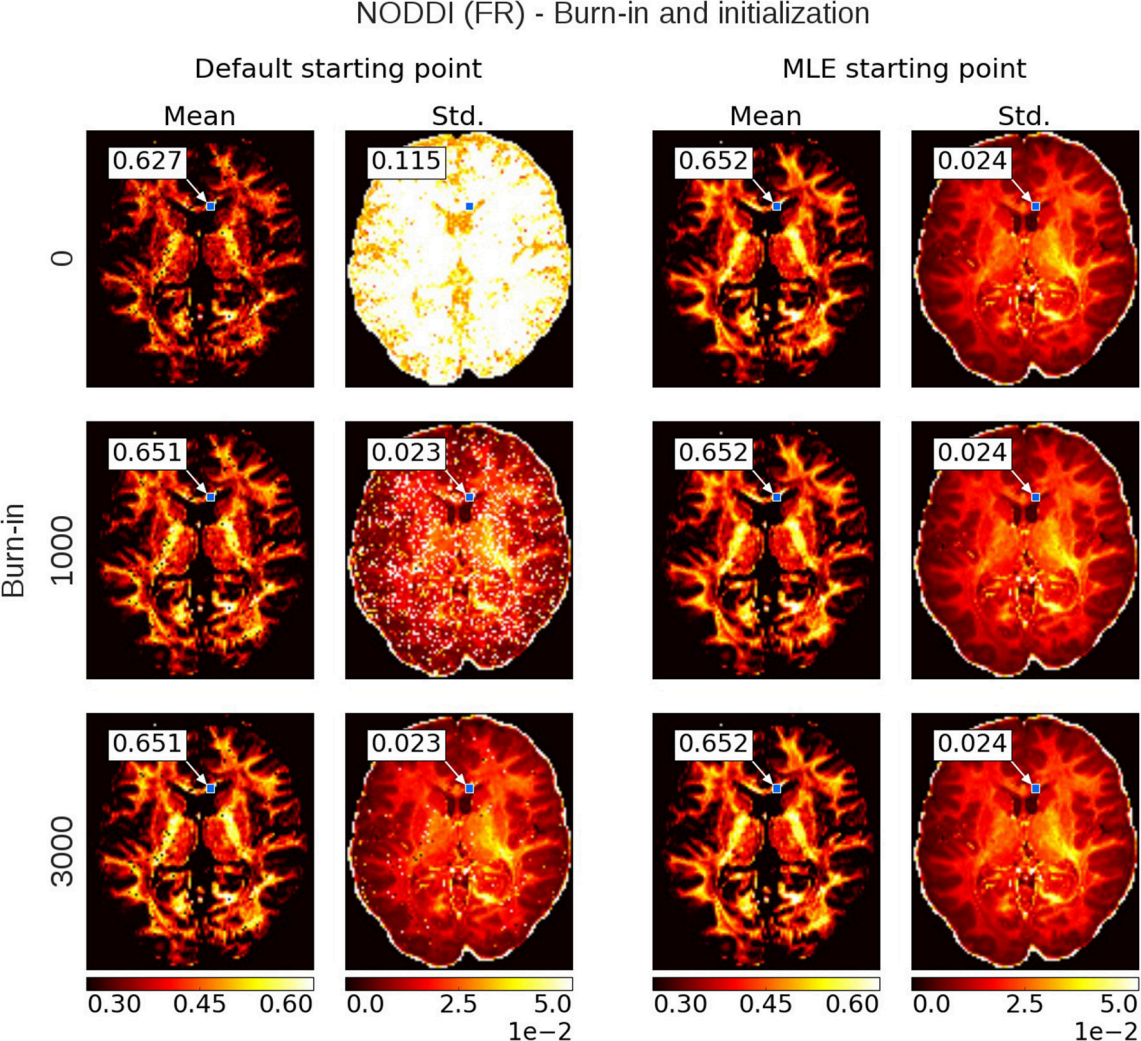
Burn-in demonstration and chain initialization using NODDI Fraction of Restricted (FR). On the left, the posterior mean and standard deviation (std.) maps when sampling NODDI from the MDT default starting point, on the right the mean and std. maps when sampling NODDI using a Maximum Likelihood Estimator (MLE) as starting point. The rows show the effect of discarding the first *z*∈{0, 1000, 3000} samples as burn-in before the mean and std. estimation. Statistics are without thinning and over 10,000 samples after *z*. The value insets show the mean and standard deviation value from a Gaussian fit to the sampling chain for the indicated voxel.

Figure 6 shows a comparison of mean and standard deviation estimates over 10,000 samples (no thinning), between sampling started from the default starting point (Appendix Table 4) and from a Maximum Likelihood Estimator starting point, over an increasing length of burn-in. When started from a default starting point, the chains of most voxels will have converged to their stationary distribution after a burn-in of about 3,000 samples. When started from an MLE starting point, the chain starts from a point in the stationary distribution and no burn-in is necessary. Starting from an MLE starting point has the additional advantage of removing salt- and pepper-like noise from the mean and std. maps. For example, even after a burn-in of 3,000 samples, there are still some voxels in the default starting point maps that have not converged yet. Burn-in also seems to have a greater impact on the standard deviation estimates than it does on the mean estimates. After a burn-in of 1,000 samples, the means of the default starting point maps seem to have converged, while the many of the standard deviations clearly have not. in contrast, stable standard deviation estimates are obtained from the MLE initialized chain even without burn-in.

**Figure 7:**
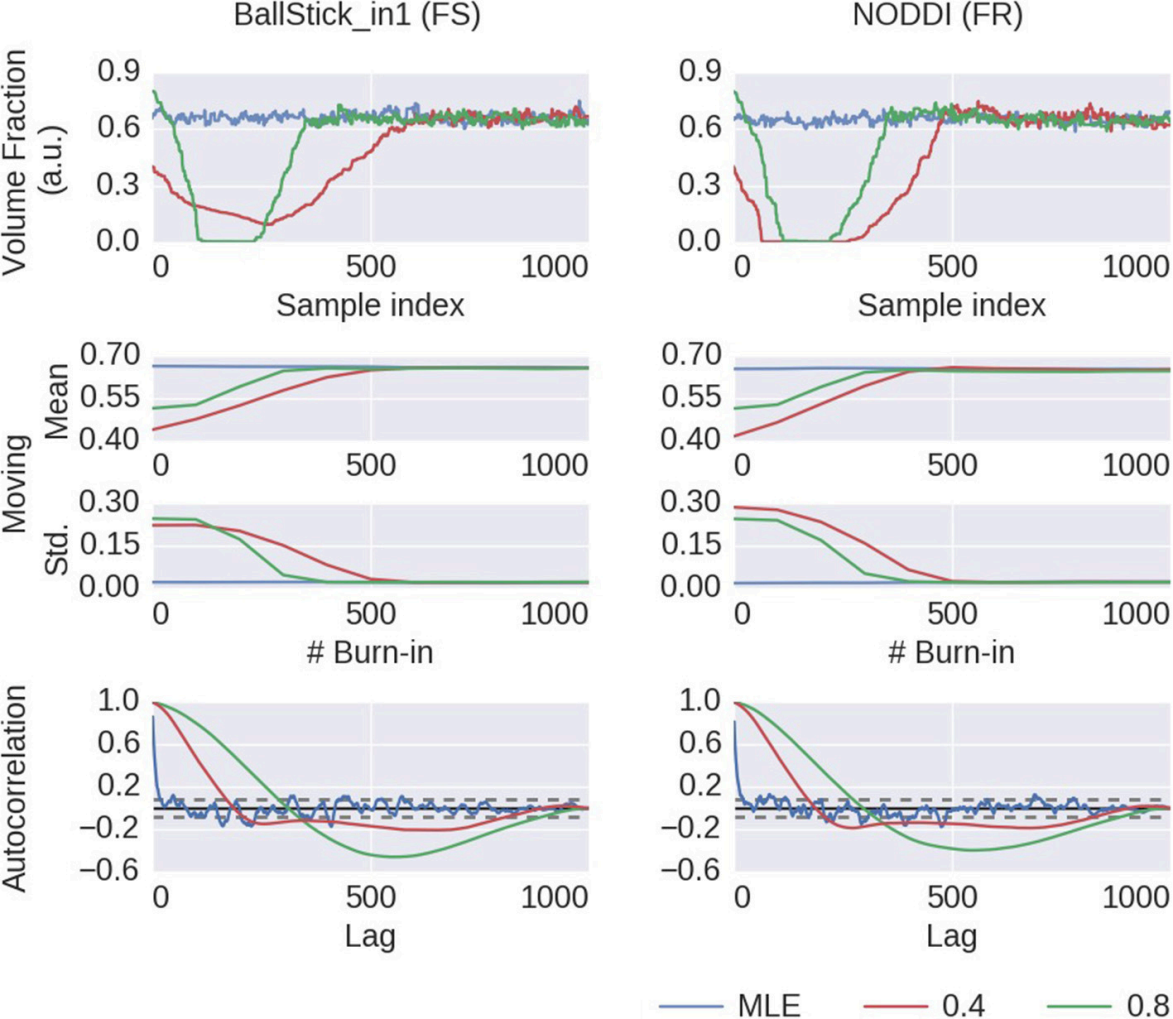
MCMC chains and burn-in results of a single voxel (the voxel indicated in Figure 6) for the BallStick_in1 Fraction of Stick (FS) and the NODDI Fraction Restricted (FR) model parameters. In the first row, the sampling trace when starting at the MLE or at two default points with (only) a varying volume fraction. In the second row, moving mean and moving standard deviations computed over 1,000 samples with increasing burn-in. In the bottom row, autocorrelation plots computed over 1,000 samples, with the 99% confidence interval in dashed gray.

To illustrate this on a single chain basis, in Figure 7 we plot the first 1,000 samples of an MCMC run of the Ball&Stick_in1 and NODDI model, for the voxel indicated in Figure 6, using the MLE starting point and two random volume fractions as a starting point, with the sampling traces in the top row showing how the sampler moves through the parameter space before converging to the stationary distribution. The second row shows the effect of discarding the first *z* samples when computing the posterior mean and standard deviation (with statistics over 1,000 samples, after the burn-in *z*), and finally in the third row autocorrelation plots for the chains of each starting point method. Interestingly, the default initialized points first seem to move toward an intra-axonal volume fraction of zero, before moving up again. This is probably caused by a misalignment of the model orientation with the data's diffusion orientation, making the intra-axonal volume less likely. Only after a correct orientation of the model, the volume fraction can go up again. The moving mean and moving standard deviation plots in the second row show the convergence of the mean and standard deviation with an increased burn-in length. These plots again show that, when started from the MLE, no burn-in is needed, while starting from another position some burn-in is required for the chains to converge. The autocorrelation also confirm that the MLE initialized chain starts from a converged state, whereas the default initialized chains are far from convergence. This chain behavior is very similar for a crossing fiber voxel, as illustrated in Supplementary Figures 3, 4.

### 3.3. Thinning

**Figure 8:**
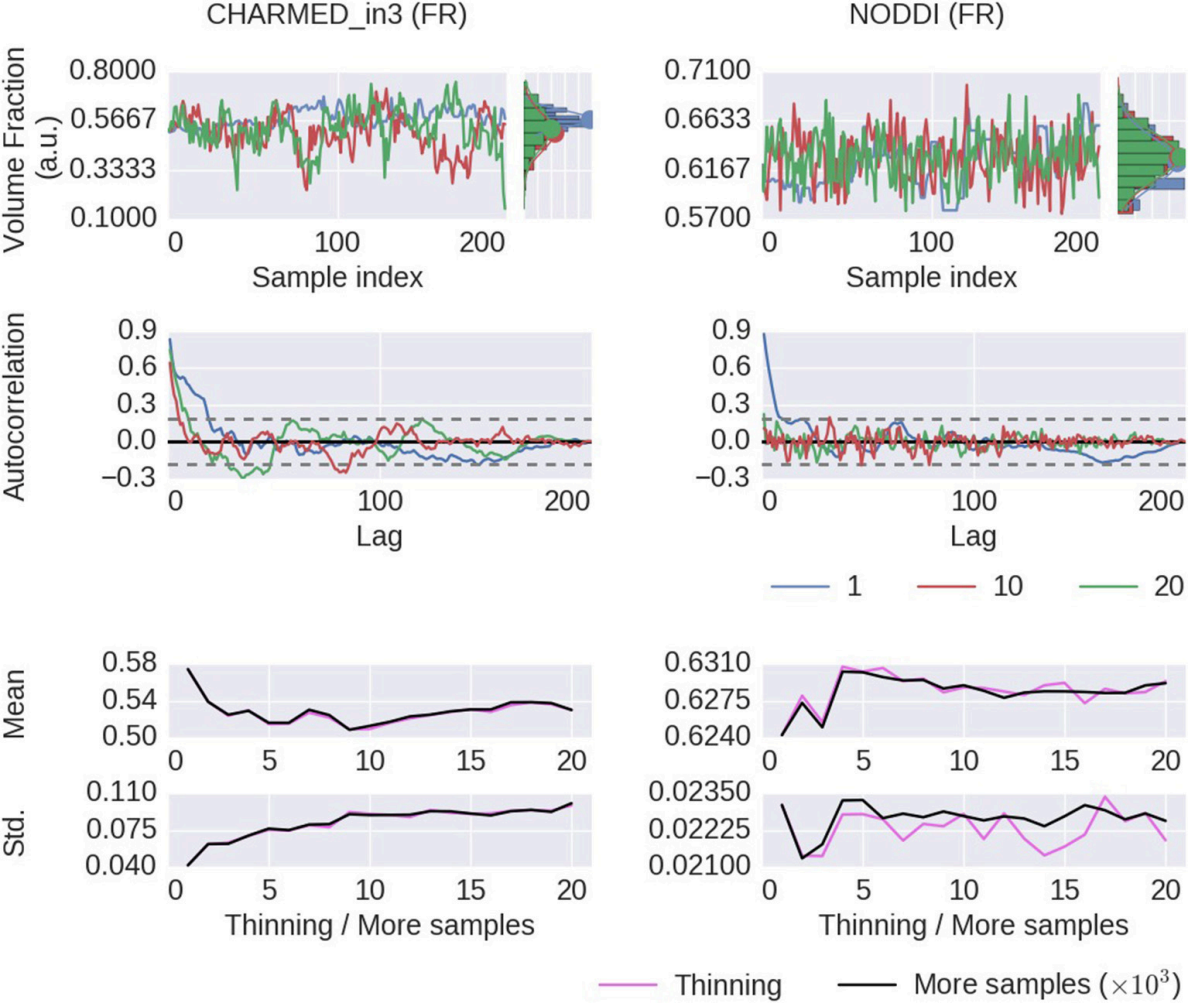
Thinning results of a single voxel (the voxel indicated in Figure 6) for the CHARMED_in3 Fraction of Restricted (FR) and the NODDI FR model parameters. In the first row, sample traces for the returned samples after a thinning of 1 (no thinning), 10 and 20, with their corresponding histograms. In the second row, an autocorrelation plot computed over 200 samples, with the 99% confidence interval in dashed gray. In the bottom row, a comparison of the posterior mean and standard deviation when thinning the chain or when using more samples. When thinning, 1000·*k* samples are generated of which only every *k*th sample is used (so, always 1,000 samples are used). When using more samples, all 1000·*k* samples are used, without thinning. Results are without burn-in and started from a maximum likelihood estimator.

Thinning of sampler chains has theoretically been shown to reduce the accuracy of posterior analyses (Geyer, 1991; MacEachern and Berliner, 1994; Link and Eaton, 2012), and empirical evidence has been provided for the limited usefulness of thinning (Link and Eaton, 2012) Here we will show some empirical results of thinning applied to diffusion MRI modeling. Figure 8 shows the effect of thinning on the variability of the returned sampling trace, on the estimates of the mean and standard deviation and in terms of autocorrelation. The sampling trace shows that the chains produce roughly the same distribution, while with increased thinning many more samples are required (*k* times more samples, for a thinning of *k*). Comparing the effect of thinning on the mean and standard deviation shows that, as predicted by theory, there is less or equal variance in the estimates when using more samples as compared to thinning the samples. Results also show that 1,000 samples without thinning may not be enough for a stable estimates and more samples are required. Yet in accordance with theory, instead of thinning the chain, results indicate that just using more samples (e.g., all 1000·*k* samples instead a thinning of *k*) is preferred. Therefore, while autocorrelations are reduced as expected for thinned chains, mean and standard deviation estimates are robust against autocorrelation and the larger number of samples without thinning is preferred over thinned samples with reduced autocorrelation. Again, behavior is reproduced for a crossing fiber voxel, as illustrated in Supplementary Figures 3, 5.

### 3.4. Minimum Number of Samples

**Figure 9:**
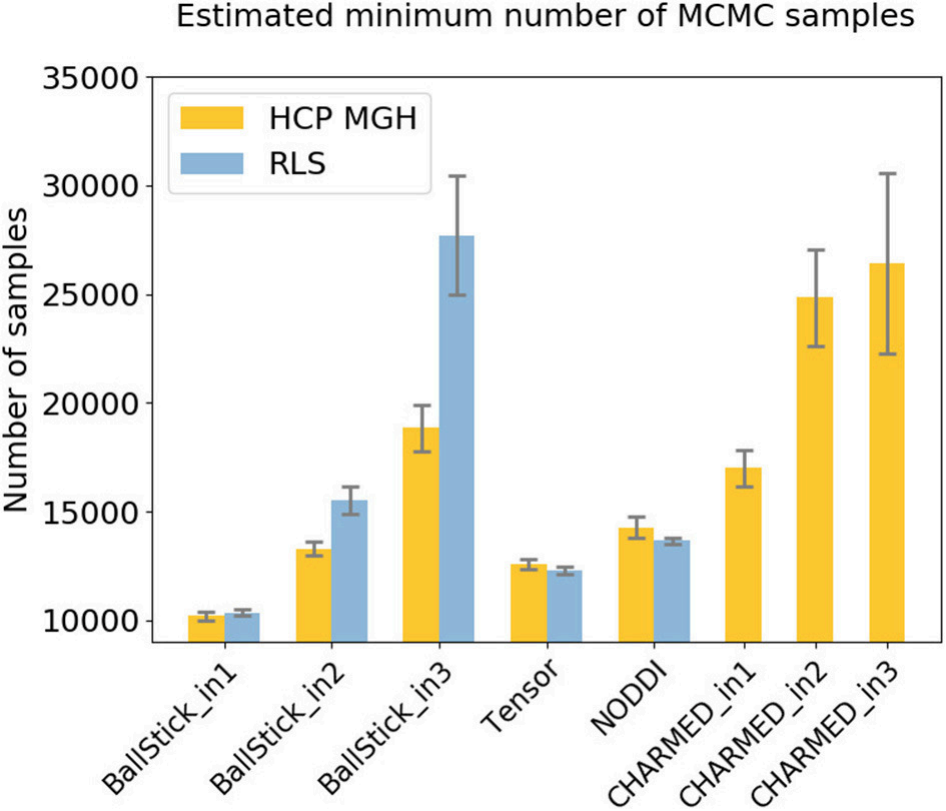
Estimates on the number of samples needed per model, to reach, when averaged over the white matter, a 95% confidence region with a 90% relative precision. Results are shown for both an HCP MGH and RLS-pilot acquisition table. Whiskers show the standard error of the mean over 10 subjects.

Using multivariate ESS theory we determined, a priori, per model, the number of actual samples needed to generate a sufficient number of effective samples (the effective sample size or ESS) to approximate the underlying posterior density within a 95% confidence region and with a 90% relative precision. Figure 9 shows an estimate on the number of actual samples needed to reach this desired ESS, on average for a large number of voxels. For lower order models (Ball&Stick_in1, Tensor, NODDI) the sampling requirements do not depend on the acquisition table, with similar numbers of samples needed for the HCP MGH and RLS-pilot protocols. For multi-directional models (Ball&Stick_in2, Ball&Stick_in3) more samples are needed for the RLS-pilot protocol than for the HCP MGH protocol where we need 1.5x to 2x the amount of samples for the RLS-pilot protocol. Since the RLS-pilot dataset is not suitable for the CHARMED models, no RLS-pilot results are shown for the CHARMED models. Two and three fiber versions of the same models (Ball&Stick and CHARMED) require almost linearly increasing number of samples. Table 5 summarizes the estimated sample requirements, as the one standard error above the mean point (upper whisker in Figure 9), together with the required ESS and the number of estimated parameters in each model. In general, models with more parameters need more actual samples to reach the same confidence and precision, although the Tensor model with seven parameters requires less samples than the NODDI model with six parameters. This is probably related to the higher complexity (non-linear parameter inter-dependencies) of the NODDI model compared to the Tensor model. As can be seen in Figure 2 (upper left panel), the required ESS to reach the desired 95% confidence region with a 90% relative precision is relatively invariant to the number of parameters (at about 2200), although the numbers of actual samples needed to realize this are different for every model, as seen in Figure 9. As an illustration of computation times, Table 6 shows runtime statistics for sampling the recommended number of samples for HCP MGH dataset and a RLS-pilot dataset using an AMD Fury X graphics card and an Intel Xeon e3 18 core processor. For most models the GPU outperforms the CPU by about 20x, except for the more complex models (CHARMED_in2, CHARMED_in3) where the GPU is only about 6x faster. In general, although 4 to 7 h are needed to sample the single fiber CHARMED_in1 and NODDI models on the very large HCP MGH dataset (with 552 volumes), GPU-accelerated implementation can provide full posterior sampling of diffusion microstructure models over whole brain datasets in reasonable time on a standard graphics card. On the more clinically feasible RLS-pilot protocol (134 volumes) whole brain sampling of Tensor and Ball&Stick models can be performed within 20 min and NODDI within an hour.

**Table 5:** Estimates on the number of samples needed per model, to reach, when averaged over the white matter, a 95% confidence region with a 90% relative precision.

**Model**	**Number of parameters**	**Required ESS**	**Required nr. of samples**
BallStick_r1	4	2,108	11,000
BallStick_r2	7	2,192	15,000
BallStick_r3	10	2,208	2,5000
NODDI	6	2,177	15,000
Tensor	7	2,192	13,000
CHARMED_r1	11	2,208	17000
CHARMED_r2	15	2,198	25,000
CHARMED_r3	19	2,183	30,000

*While the required ESS can be determined a priori (see Figure 9), the inherent model complexity determines how many samples are needed to reach that ESS*.

**Table 6:** Runtime statistics in hours (h) for MCMC sampling the estimated minimum number of samples (no burn-in, no-thinning) of various models, using a single HCP MGH (552 volumes) and single RLS-pilot (134 volumes) dataset.

**Model**	**Number of samples**	**HCP MGH - GPU**	**HCP MGH - CPU**	**RLS-pilot - GPU**	**RLS-pilot - CPU**
Ball&Stick_in1	11,000	1 h	36 h	0.25 h	4.5 h
Ball&Stick_in2	15,000	2 h	48 h	0.5 h	15 h
Ball&Stick_in3	25,000	6 h	72 h	1.5 h	45 h
NODDI	15,000	4 h	80 h	1 h	20 h
Tensor	13,000	1 h	22 h	0.5 h	4 h
CHARMED_in1	17,000	6 h	54 h	n/a	n/a
CHARMED_in2	25,000	24 h	139 h	n/a	n/a
CHARMED_in3	25,000	47 h	200 h	n/a	n/a

*Statistics are for a whole brain mask of 410,000 voxels for HCP MGH and 204,993 voxels for RLS-pilot. Results are computed using an AMD Fury X GPU and an Intel Xeon e3 18 core CPU*.

## 4. Discussion

Using an efficient GPU based implementation, we show that run times can be removed as a prohibitive constraint for sampling of diffusion multi-compartment models, achieving whole brain sampling in under an hour for typical datasets and most common dMRI models. Newer generations of graphics cards are likely to reduce these times even further. Using this implementation, we investigated the use of adaptive MCMC algorithms, burn-in, initialization, and thinning. We finally applied the theory of Effective Sample Size to diffusion multi-compartment models as a way of determining a sufficient number of samples for a given model and dataset.

### 4.1. Adaptive MCMC

The use of adaptive MCMC algorithms increases the effectiveness of the sampling process by generating more effective samples for the same amount of MCMC samples. Adaptative methods generally have higher multivariate Effective Sample Size (ESS) than MCMC without adaptation. Although adaptative methods generally score close to each other in generated ESS, the FSL, and AMWG methods have better sampling efficiency for complex crossing fiber models, such as CHARMED_in3 and low #volumes/#parameters situations, with AWMG slightly outperforming FSL. In accuracy and precision, AMWG and FSL perform well overall, whereas performance for the fixed proposal method (None) and SCAM is more inconsistent over all models. The ESS performance of the fixed proposal method could, in theory, be increased to the same levels as the adaptive methods by manual calibration, but since this is model and data (voxel) dependent, manual tuning could be very burdensome and unpractical. This work covers only variations of the Metropolis-Within-Gibbs method, which has the advantage of high efficiency sampling with relatively general model-unspecific proposals. Future work could focus on MCMC algorithms which allow for block-updates of correlated parameters, or could investigate different proposal schemes altogether such as Component-wise Hit-And-Run Metropolis (Turchin, 1971; Smith, 1984), Multiple-Try Metropolis (Liu et al., 2000) and/or No-U-Turn sampler (Hoffman and Gelman, 2011).

### 4.2. Burn-in

When starting from an arbitrary position, burn-in is advisable to reduce possible bias due to (possibly) low probability starting positions. Burn-in should ideally be considered post-sampling, since it is difficult to know a priori the time needed for the chain to converge and, due to randomness, past convergence rates provide no guarantee for the future. This is why common practice dictates a relatively large number of burn-in samples which guarantees convergence in most cases.

While not harmful, burn-in is generally unnecessary and inefficient if the starting point is part of the stationary distribution of the Markov chain, which can, for example, be achieved by taking a Maximum Likelihood Estimator (MLE) as starting point. Even when starting from an MLE, a small burn-in of about 100 to 200 samples could be considered to remove correlations with the starting position. Additionally, when using adaptive proposal methods, a small burn-in could be considered to let the adaptation algorithm adapt the proposal distribution before sampling, slightly increasing the effective sample size of the chain.

### 4.3. Thinning

Already on theoretical grounds, thinning is not recommended and considered as often unnecessary, always inefficient and reducing the precision of posterior estimates (Geyer, 1991; MacEachern and Berliner, 1994; Jackman, 2009; Christensen et al., 2010; Link and Eaton, 2012). Illustrations based on the the Ball&Stick_in1 and NODDI model show that, with or without thinning, the posterior distribution is approximated about equally, while thinning needs *k* times more samples (for a thinning of *k*). Results did show a convergence of mean and standard deviation estimates with an increased thinning, but these results are easily duplicated by incorporating not only the thinned samples but also the non-thinned samples in the statistical estimates (the “more samples” strategy). Furthermore, using more samples instead of thinning provides estimates with a higher precision, as illustrated by the higher variability of the thinned estimates compared to the estimates with more samples (Figure 8, right). One legitimate reason for thinning is that, with independent samples, one can approximate the precision of an MCMC approximation (Link and Eaton, 2012). That is, it allows for more accurate assessment of the standard error of an MCMC estimate like the posterior mean. However, even in that case, thinning must be applied *post-hoc*, otherwise the precision of the mean itself will be reduced if computed from only the thinned samples. Furthermore, we are often more interested in the variability of the posterior distribution (which can be provided by e.g., the standard deviation) than in the precision of the posterior mean estimate. Another legitimate reason for considering thinning is hardware limitations, such as sampling post-processing time and storage space. However, barring such limitations, avoiding thinning of chains is far more efficient in providing high precision in posterior estimates.

### 4.4. Number of Samples

The issue of the number of samples needed in a chain is often somewhat enigmatic and arbitrary. A common perception is that the number should be “high,” rather too high than too low. Multivariate Effective Sample Size (ESS) theory provides a theoretical lower bound on the number of effective samples needed to approximate the posterior, based on a desired confidence level and precision. How many MCMC samples are required to reach that target effective sample size is then dependent on the data, the model and the MCMC algorithm. We show that there is some dependency on the data in terms of sampling requirements for diffusion microstructure models, considering the increasing discrepancy between required sample numbers for Ball&Stick_in1, Ball&Stick_in2, and Ball&Stick_in3. The dependency of sampling requirements on the model is higher, showing that more complex models seem to need more actual samples to reach the target ESS. As can be seen in Figure 2 (upper left panel), the required ESS to reach the 95% confidence region with a 90% relative precision is relatively invariant to the number of parameters (at about 2200), although the numbers of actual samples needed to realize this are different for every model, as seen in Figure 9. This sets an informed relatively general target for the amount of samples required in sampling diffusion micro-structural models, which scales the number of actual samples with the complexity of the model, data, and the performance of the MCMC algorithm. This also means that MCMC algorithms which can generate effective samples more efficiently (such as the AMWG) can reduce the number of samples needed to reach the same confidence levels, reducing run-time.

## 5. Conclusions and Recommendations

Considering the theoretical soundness and its general robust performance, we advise to use the Adaptive Metropolis-Within-Gibbs (AMWG) algorithm for efficient and robust sampling of diffusion MRI models. We further recommend initializing the sampler with a maximum likelihood estimator obtained from, for example, non-linear optimization, in which case 100 to 200 samples are sufficient as a burn-in. Thinning is unnecessary unless there are memory or hard disk constraints or a strong reliance on posterior estimates that require uncorrelated samples. As a relatively general target for the number of samples, we recommend 2,200 multivariate effective samples, which reaches 95% confidence and 90% relative precision, invariant of the number of parameters. The amount of actual MCMC samples required to achieve this is algorithm and model dependent and can be investigated in a pre-study, with numbers for common dMRI models reported here as an indication.

## Author Contributions

RH and AR contributed conception and design of the study. RH wrote the software and performed the analysis. RH wrote the first draft of the manuscript. RH and AR wrote sections of the manuscript. All authors contributed to manuscript revision, read and approved the submitted version.

### Conflict of Interest Statement

The authors declare that the research was conducted in the absence of any commercial or financial relationships that could be construed as a potential conflict of interest.
