# Supplementary material for: Robust and Fast Markov Chain Monte Carlo Sampling of Diffusion MRI Microstructure Models
Source: Front Neuroinform. 2018 Dec 18;12:97. doi: 10.3389/fninf.2018.00097 (PMC6305549; doi:10.3389/fninf.2018.00097)
Supplement: Supplementary file 1 [file Data_Sheet_1.PDF]

# Supplementary material

to the article

# Robust and fast Monte Carlo Markov Chain sampling of diffusion MRI microstructure models

R.L. Harms[a], A. Roebroeck[a]

[a]*Dept. of Cognitive Neuroscience, Faculty of Psychology & Neuroscience, Maastricht University, the Netherlands*

## 1 Adaptive proposal strategies

The following figures are similar in structure to the corresponding figures in the article, except they use twice the amount of samples. That is, these figures are based on 20000 samples, with the first 10000 samples equal to the samples in the article. Supplementary figure 1 is equal to article figure 4 and supplementary figure 2 corresponds to article figure 5.

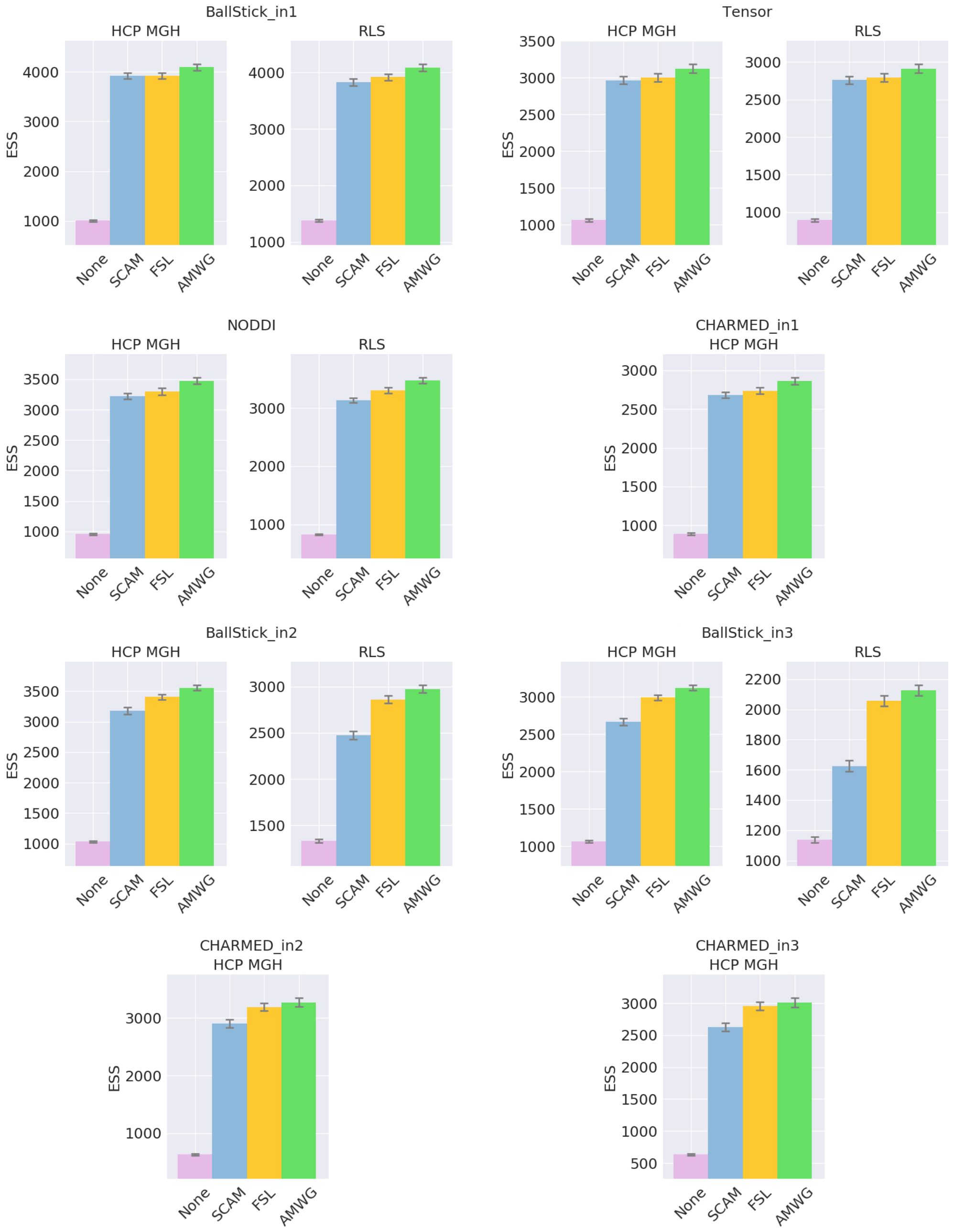


Figure 1: Estimated multivariate Effective Sample Size (ESS), for no adaptive metropolis (None), the Single Component Adaptive Metropolis (SCAM), the FSL acceptance rate scaling (FSL) and Adaptive Metropolis-Within-Gibbs (AMWG) adaptive proposal methods. Whiskers show the standard error of the mean computed over 10 repeats. Results are over 20000 samples, without burn-in and thinning.

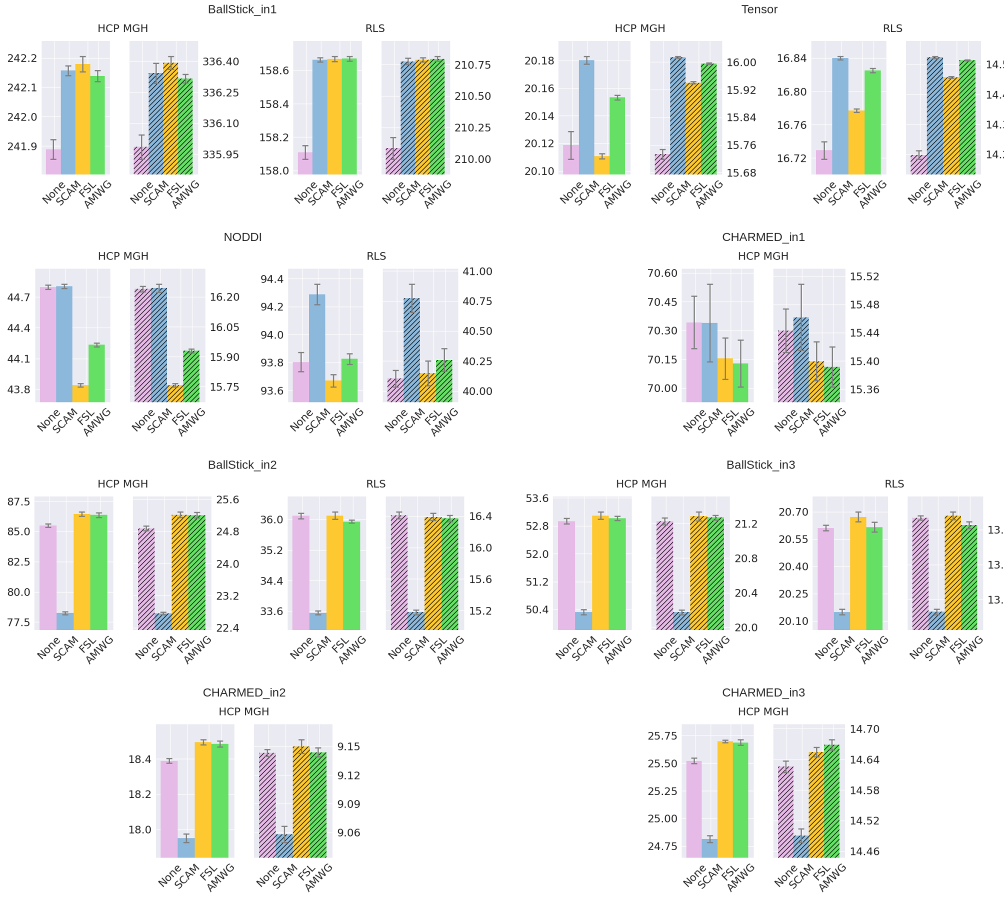


Figure 2: Estimated accuracy (left plots) and precision (right, shaded, plots), for no adaptive metropolis (None), the Single Component Adaptive Metropolis (SCAM), the FSL acceptance rate scaling (FSL) and Adaptive Metropolis-Within-Gibbs (AMWG) adaptive proposal methods. The results are averaged over 10000 voxels and 10 trials, the whiskers show the standard error of the mean computed over the 10 trials. Results are over 20000 samples, without burn-in and thinning.

## 2 Burn-in and thinning

Burn-in and thinning demonstration on a voxel which is anticipated to have multiple directions.

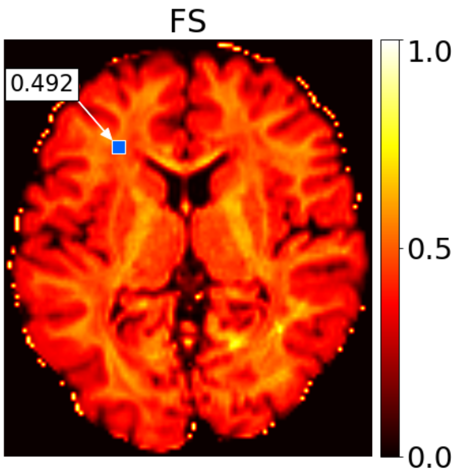


Figure 3: The voxel used for the results in figure S4

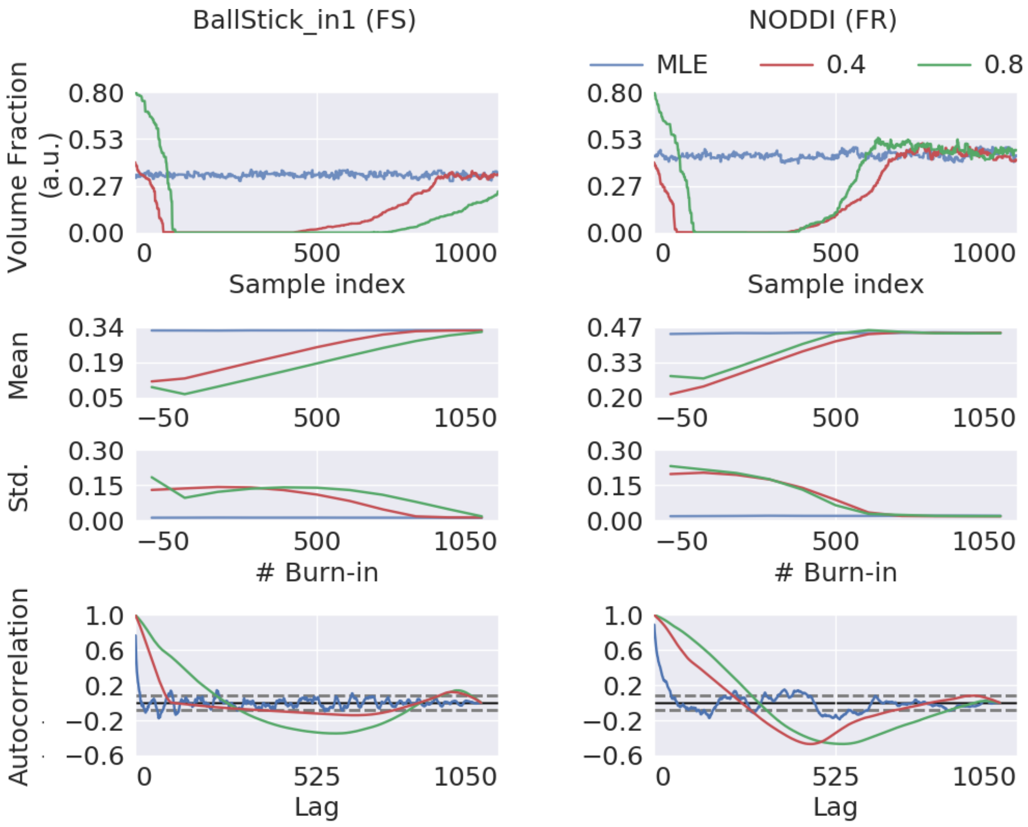


Figure 4: MCMC chains and burn-in results of a single voxel (the voxel indicated in supplementary figure 3) for the BallStick_in1 Fraction of Stick (FS) and the NODDI Fraction Restricted (FR) model parameters. In the first row, the sampling trace when starting at the MLE or at two default points with (only) a varying volume fraction. In the second row, moving mean and moving standard deviations computed over 1000 samples with increasing burn-in. In the bottom row, autocorrelation plots computed over 1000 samples, with the 99% confidence interval in dashed gray.

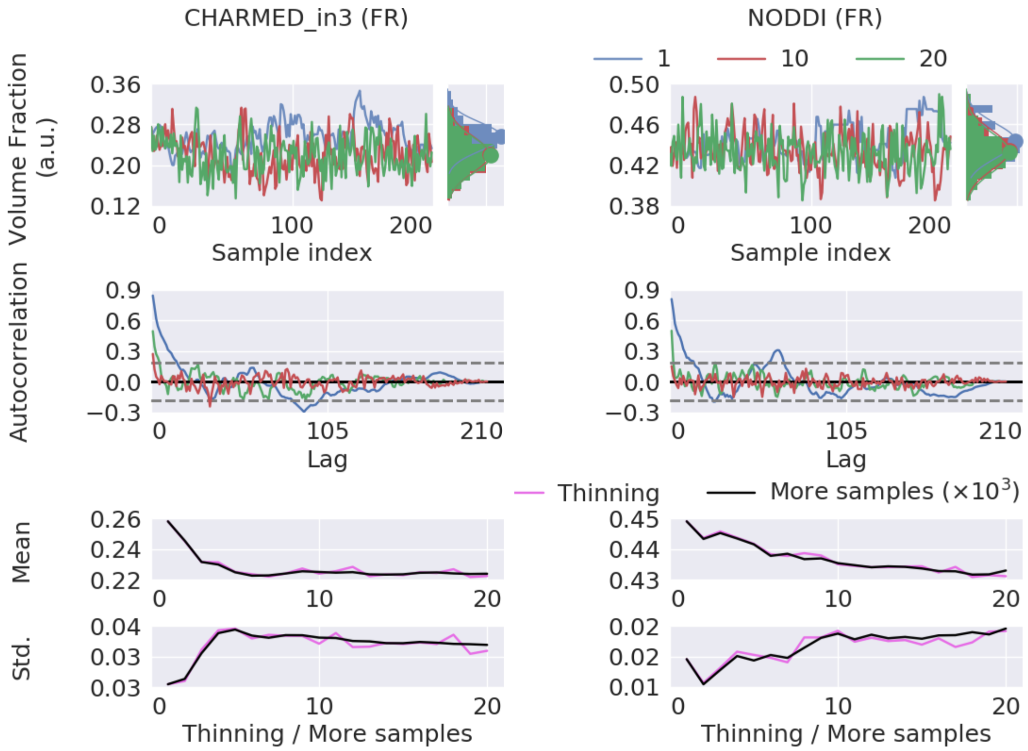


Figure 5: Thinning results of a single voxel (the voxel indicated in figure 3) for the CHARMED_in3 Fraction of Restricted (FR) and the NODDI FR model parameters. In the first row, sample traces for the returned samples after a thinning of 1 (no thinning), 10 and 20, with their corresponding histograms. In the second row, an autocorrelation plot computed over 200 samples, with the 99% confidence interval in dashed gray. In the bottom row, a comparison of the posterior mean and standard deviation when thinning the chain or when using more samples. When thinning, $1000 \cdot k$ samples are generated of which only every $k$th sample is used (so, always 1000 samples are used). When using more samples, all $1000 \cdot k$ samples are used, without thinning. Results are without burn-in and started from a maximum likelihood estimator.
