# Supplementary material for: Robust and Fast Markov Chain Monte Carlo Sampling of Diffusion MRI Microstructure Models
Source: Front Neuroinform. 2018 Dec 18;12:97. doi: 10.3389/fninf.2018.00097 (PMC6305549; doi:10.3389/fninf.2018.00097)
Supplement: Supplementary file 2 [file Data_Sheet_2.pdf]

Appendix

to the article

Robust and fast Monte Carlo Markov Chain sampling of diffusion MRI microstructure models

R.L. Harms[a], A. Roebroeck[a]

[a]*Dept. of Cognitive Neuroscience, Faculty of Psychology & Neuroscience, Maastricht University, the Netherlands*

1 MCMC priors

The priors used during MCMC sampling.

**Parameter**	**Prior**
S_0	$U(0, 1 \cdot 10^{10})$
w_i	$U(0, 1)$
$d_{\parallel}$ (or d)	$U(3 \cdot 10^{-11}, 1 \cdot 10^{-8})$
$d_{\perp_1}$, $d_{\perp_2}$	$U(0, 1 \cdot 10^{-8})$
θ, ϕ	$U(0, \pi)$
ψ	$U(0, \pi)$
κ	$U(0, 2\pi)$

Table 1: The priors $p_i(\mathbf{x}_i)$ per model parameter. These priors are combined with the model specific parameters in table 2 to form the complete model priors. For parameter usage and specification see table 3 in the article.

**Model (M)**	**Prior**
BallStick_in[n], NODDI, CHARMED_in[n]	$p_j(\mathbf{x}, M) = 1$ if $\sum_{k=0}^{n-1} \mathrm{w}_k <= 1$, else $p_j(\mathbf{x}, M) = 0$
Tensor, CHARMED_in[n] (extra axonal compartment)	$p_j(\mathbf{x}, M) = 1$ if $d > d_{\perp_0} > d_{\perp_1}$, else $p_j(\mathbf{x}, M) = 0$

Table 2: The model priors $p_j(\mathbf{x}|M)$ for model M. Each of these priors should be interpreted as a boolean, that is, they return a value of 1 if the condition is fulfilled, else they return 0. These priors are combined with the parameter specific priors in table 1 to form the complete model priors.

2 MCMC proposal distributions

The default proposal distributions used in MCMC sampling.

Parameter	Proposal distribution
S_0	$\mathcal{N}(\mathbf{X}_i^{(t)}, 10)$
w_i	$\mathcal{N}(\mathbf{X}_i^{(t)}, 0.01)$
d, $d_{\parallel}$, $d_{\perp_1}$, $d_{\perp_2}$	$\mathcal{N}(\mathbf{X}_i^{(t)}, 1 \cdot 10^{-10})$
ψ	$\mathcal{N}(\mathbf{X}_i^{(t)}, 0.1) \mod \pi$
θ, ϕ	$\mathcal{N}(\mathbf{X}_i^{(t)}, 0.1)$
κ	$\mathcal{N}(\mathbf{X}_i^{(t)}, 0.01)$

Table 3: The proposal distributions $q_i(\mathbf{X}^{(t+1)^*}|\cdot)$ per model parameter with their default proposal standard deviations. For parameter usage and specification see table 3 in the article.

3 Default starting point

The following table lists the default initialization point when not initializing with an Maximum Likelihood Estimator.

Parameter	Default starting value
S_0	$1 \cdot 10^4$
w_i	0.5
$d_\parallel$ (or d)	$1.7 \cdot 10^{-9}$
$d_{\perp_1}$	$1.7 \cdot 10^{-10}$
$d_{\perp_2}$	$1.7 \cdot 10^{-11}$
θ, ϕ, ψ	$\pi/2$
κ	1

Table 4: The default starting points for the MCMC sampler, used only when the sampler is not initialized with a maximum likelihood estimator.
